# The transition from local to global patterns governs the differentiation of mouse blastocysts

**DOI:** 10.1371/journal.pone.0233030

**Published:** 2020-05-15

**Authors:** Sabine C. Fischer, Elena Corujo-Simon, Joaquin Lilao-Garzon, Ernst H. K. Stelzer, Silvia Muñoz-Descalzo

**Affiliations:** 1 Physikalische Biologie, Buchmann Institute for Molecular Life Sciences, Goethe-Universität Frankfurt am Main, Frankfurt am Main, Germany; 2 Department of Biology and Biochemistry, University of Bath, Bath, England, United Kingdom; 3 Instituto Universitario de Investigaciones Biomédicas y Sanitarias, Universidad Las Palmas de Gran Canaria, Las Palmas de Gran Canaria, Las Palmas de Gran Canaria, Spain; Western University, CANADA

## Abstract

During mammalian blastocyst development, inner cell mass (ICM) cells differentiate into epiblast (Epi) or primitive endoderm (PrE). These two fates are characterized by the expression of the transcription factors NANOG and GATA6, respectively. Here, we investigate the spatio-temporal distribution of NANOG and GATA6 expressing cells in the ICM of the mouse blastocysts with quantitative three-dimensional single cell-based neighbourhood analyses. We define the cell neighbourhood by local features, which include the expression levels of both fate markers expressed in each cell and its neighbours, and the number of neighbouring cells. We further include the position of a cell relative to the centre of the ICM as a global positional feature. Our analyses reveal a local three-dimensional pattern that is already present in early blastocysts: 1) Cells expressing the highest NANOG levels are surrounded by approximately nine neighbours, while 2) cells expressing GATA6 cluster according to their GATA6 levels. This local pattern evolves into a global pattern in the ICM that starts to emerge in mid blastocysts. We show that FGF/MAPK signalling is involved in the three-dimensional distribution of the cells and, using a mutant background, we further show that the GATA6 neighbourhood is regulated by NANOG. Our quantitative study suggests that the three-dimensional cell neighbourhood plays a role in Epi and PrE precursor specification. Our results highlight the importance of analysing the three-dimensional cell neighbourhood while investigating cell fate decisions during early mouse embryonic development.

## Introduction

During mammalian preimplantation development, two sequential cell fate decisions occur that result in three cell populations (reviewed in [1]). Upon the first decision, cells become either trophectoderm (TE) or inner cell mass (ICM) cells. Descendants of TE cells form the foetal portion of the placenta. The ICM cells make a further decision: they differentiate either into Epiblast (Epi) or into Primitive Endoderm (PrE). Epi cells predominantly give rise to the embryo proper while PrE cell descendants mainly generate the endodermal part of the yolk sac. In mice, three major processes have been proposed for ICM cell differentiation into Epi or PrE [2,3]. In early blastocysts, ICM cells co-express Epi and PrE markers such as NANOG and GATA6, respectively. As time progresses, Epi and PrE progenitors arise. Epi progenitors express high levels of NANOG, and almost no GATA6, while PrE progenitors express high levels of GATA6, and almost no NANOG [3–5]. FGF/MAPK signalling reinforces PrE commitment: Epi progenitors secrete FGF4, which binds to FGFR1 on Epi, and FGFR1 and FGFR2 on PrE biased cells [4,6–11]. This results in a distribution of Epi and PrE progenitors in the ICM without an obvious spatial pattern [3,12,13]. As development progresses, PrE progenitors migrate to occupy their position before the embryo implants. This results in the spatial segregation of the two lineages. PrE progenitors are polarized and positioned at the surface of the ICM, where they form an epithelium in contact with the blastocyst cavity or blastocoele [14–16]. The Epi progenitors occupy a central position between TE and PrE.

Epi versus PrE differentiation has been extensively studied in the context of marker expression dynamics and the involved signalling pathways (reviewed in [1]). Technical developments have made it possible to study cell fate decisions during preimplantation mouse development at single-cell resolution (reviewed in [17]). Invasive studies based on single cell transcriptomics have been used. However, transcriptomic techniques disrupt the cell positional information within the ICM [8,9,11,18,19]. A complementary approach is single cell resolution imaging based on immunofluorescence stainings [4–6,9,13,20–22] or fluorescent reporters [10,23–32]. Combined with quantitative image analysis, the immunofluorescence approach provides protein expression levels together with cell positional information. Applying this technique in our recent study in mouse embryos and ICM organoids has revealed a local cell fate clustering during PrE differentiation [12].

Here, we broaden the three-dimensional analysis of the spatial distribution of NANOG and GATA6 expressing cells in the ICMs of mouse embryos at different stages. We combine the positional information of a cell and its expression levels of NANOG and GATA6 with information of its neighbouring cells to obtain the local cell neighbourhood features and a global positional feature (see Terminology Box). We find a complex pattern in the three-dimensional cell neighbourhood in the ICM of early blastocysts, characterized by local positional features and expression level clusters (see Terminology Box). Highest NANOG expression levels are found in cells with a specific number of neighbours. The GATA6 level of a cell correlates with the levels of GATA6 in its neighbours, resulting in GATA6 expression level clusters. We apply a rule-based computer simulation to show that these two local cell neighbourhood features are sufficient to describe the complex population distribution found in early embryos. We further demonstrate that the simulations are also applicable in a *Nanog* mutant background. As potential regulators of the expression level clustering, we identify NANOG and FGF/MAPK signalling. Patterns in the global positional features start in mid blastocyst and are obvious in late blastocysts with *Nanog* regulating this feature in GATA6 expressing cells.

**Table:** Terminology box.

**General terms**	
Cell neighbourhood	Cell vicinity as determined by the Delaunay Cell Graph, this implies that cells in direct contact or at close distance are neighbours (max. 30 μm)
Feature	Measurement used to describe a specific trait (e.g.: the expression level of a fate marker in a cell)
Pattern	Non-random spatial distribution
**Measurements to describe the expression pattern in ICM**	
Expression levels	NANOG or GATA6 mean fluorescence intensity per nucleus; results in continuous values
Cell state	N+, N-, G+, G- as discrete categories
Population type	Double negative (DN), double positive (DP), NANOG positive and GATA6 negative (N+G-), NANOG negative and GATA6 positive (N-G+), as discrete categories
Cell fate	Epi or PrE
Local cell features	Measurements for an individual cell related to: Positional information (cell nucleus centroid) Continuous expression levels of NANOG and GATA6 Discrete population type Number of neighbours
Local cell neighbourhood	Cells and neighbouring cells (determined by DCG) with all their features
Local positional feature	Fate marker expression levels in a cell vs number of its neighbours
Local expression level feature	Fate marker expression levels in a cell vs fate marker expression levels of its neighbours
Population cluster	Group of neighbouring cells of the same population (discrete clustering, e. g. N+G6- cells in early embryos)
Expression level cluster	Group of neighbouring cells with correlated levels of a fate marker (continuous clustering; e.g. DP cells according to their GATA6 expression levels)
Local cell neighbourhood features	Description of the local cell neighbourhood including: - Local positional feature - Local expression level feature - Population cluster - Expression level cluster
Global positional feature	Expression levels of a cell relative to its distance from the centroid of the ICM

In summary, we present three-dimensional cell neighbourhood analyses that allow a novel approach in the study of Epi versus PrE differentiation in relation to nearby cells and fate marker expression levels. Our results point at NANOG and FGF/MAPK-dependent mechanisms as responsible for the spatial arrangement of NANOG and GATA6 expressing cells in the ICM. These mechanisms become obvious in local cell neighbourhood features. Importantly, we present for the first time a signature that correlates with Epi precursor specification.

## Materials and methods

### Ethics statement

Mouse work was approved by the University of Bath Animal Welfare and Ethical Review Body (AWERB) and undertaken under UK Home Office license PPL 30/3219 in accordance with the Animals (Scientific Procedures) Act incorporating EU Directive 2010/63/EU. Additional mouse work was approved by the Consejería de Agricultura, Ganadería, Pesca y Aguas of the Gobierno de Canarias (CEEA-ULPGC 08/2018).

### Mice, embryos and immunohistochemistry

Wild-type CD1 and *Nanog*^*+/+*^, *Nanog*^*+/-*^ and *Nanog*^*-/-*^ embryos were generated by in-house breeding and natural mating. Detection of copulation plug confirmed successful mating; the resulting embryos were then considered embryonic day (E) 0.5. Embryos were isolated in M2 medium (Millipore). Embryos were prepared for immunofluorescence as previously described (89). Primary antibodies used were: anti-NANOG (eBiosciences 14–5761, 1:100), anti-GATA6 (R&D, AF1700, 1:200). Nuclei were stained using DAPI or Hoechst (1:1000, Invitrogen). Embryos were mounted on microscopy slides with Vaseline bridges to prevent their crushing. Three independent immunofluorescence stainings, each with E3.5 and E4.5 embryos from 7 litters, were performed for the first wild-type data set.

*Nanog* mutant embryos were obtained as previously described [4] and genotyped by NANOG antibody staining.

### Imaging and automated image analysis

For the first wild-type data set (S1 and S2 Tables, data I) a total of 45 embryos was imaged in four batches of 19, 15, 2 and 9 embryos. Images were acquired using a Zeiss LSM 510-META and a Plan-Apochromat 63x/1.4 Oil Ph3 objective, with optical section thickness of 1 μm. *Nanog* wild-type, heterozygous and mutant embryos (S1 and S2 Tables, data III and IV) were similarly imaged in 5 confocal sessions using a Zeiss LSM700 and a Plan-Apochromat 40x/1.3 Oil DIC (UV) VIS-IR M27 objective. All images in each imaging session were obtained using the sequential scanning mode, with the same conditions of laser intensity, gain, and pinhole, and were processed in exactly the same way. The range indicator palette option (Zeiss AIM/ZEN software) was used to ensure that no oversaturated images were taken. For a schematic representation of the image and data pre-processing and further analysis, see also S1 Fig. The three-dimensional image stacks were segmented using MINS [33], cells were automatically assigned to ICM or TE, the features of the cell nuclei were extracted including the nuclear centroid and volume, together with the mean intensity of NANOG and GATA6 for each nucleus. The automatically assigned TE or ICM fate was manually checked (S1 Fig Step1). Given the extension of the analysed data sets (over 27.000 cells) a manual correction of the segmentation results was not performed. Extreme errors (over-segmentation and pyknotic nuclei) in the segmentation were removed manually when correcting the classification of TE versus ICM.

### Data analysis

The calculations were performed with *Mathematica* 11.1 (Wolfram Research). Details on the total number of embryos and cells in each population type analysed are shown in S1 and S2 Tables. For further details, see S1 Text and S1 Fig.

### Pre-processing and staging of data from [22] (data II)

We used the embryos labelled as “littermate”, available from GitHub [22]. This resulted in 147 additional data sets (S1 and S2 Tables, data II). Compared to data I, the experimental setup was slightly different. Specifically, a different NANOG antibody was used and the embryos were imaged without being mounted. Given the extra thickness of the samples, the correction of fluorescent decay along the z-axis was required. Furthermore, while the same algorithm was used for the segmentation, a different thresholding method was applied to obtain the four populations (k-means clustering). We used the same cell number-based staging method as the one used for our data set, which resulted in 64 early, 34 mid and 49 late blastocysts. We excluded all NANOG and GATA6 levels from the distribution that were two standard deviations away from the respective mean as we noticed that there were some oversaturated nuclei images. The calculations were performed with *Mathematica* 11.1 (Wolfram Research). For further details, see S1 Text.

### Cell graph generation and neighbourhood analyses

We derived a cell graph representation to characterize the spatial distribution of the cells in each embryo in our wild-type data set (data I), 73 *Nanog*^*+/+*^ or *Nanog*^*+/-*^ embryos (data III) and 19 *Nanog*^*-/-*^ (data IV), and the data set from [22] (data II) (S1 Fig, Step 3(ii)).

The calculations were performed with *Mathematica* 11.1 (Wolfram Research). For further details, see S1 Text.

### Correlation analyses

The Spearman’s correlation analysis and bootstrapping were performed in Matlab R2012b (MathWorks). The simulations of the null model were performed with *Mathematica* 11.1 (Wolfram Research).

To classify the strength of the correlations we used the criteria by Evans [34]:

0.00–0.19: ‘very weak’
0.20–0.39: ‘weak’
0.40–0.59: ‘moderate’
0.60–0.79: ‘strong’
0.80–1.0: ‘very strong’.

For further details, see S1 Text.

### Analyses of global positional feature

We aimed to investigate global patterns within the ICM at all three blastocysts stages. To this end, a reference point is needed. The one that immediately springs to mind is the embryo centroid. However, distributions of cells with respect to the embryo centroid would mainly highlight effects from the sorting process in mid and late embryos. Therefore, we decided to use the ICM centroid as reference point and analysed the expression levels of NANOG and GATA6 with respect to a cell’s distance to the ICM centroid. The ICM centroid was determined as the mean of the positions of all ICM cells. For the graphs, the distance in μm was binned into 5 μm intervals, which is the typical radius of an ICM cell.

### Rule-based simulations of population composition in ICM of early blastocysts

The calculations were performed with *Mathematica* 11.1 (Wolfram Research). For further details, see S1 Text.

For the simulations shown in Fig 7D (*Nanog* mutant embryos), we use the cell positions, cells proportions from early *Nanog*^*+/+*^ or *Nanog*^*+/-*^ embryos (44 embryos; *p*_*GATA*6_ = 94% and *p_NANOG_* = 78%) and *startNumNeigh* = 9 to simulate the wild-type situation. For the *Nanog* mutants, we set the proportion of N+ cells (*p_NANOG_*) to 0.

### Statistics

For the comparison of expression levels, Mann-Whitney tests were applied as the distribution do not follow a Gaussian (performed in Matlab [35]). To compare populations proportions, z-tests with Bonferroni corrections were applied. The used statistical test is indicated in the figure legends.

### Data accessibility

For the segmentation and the Delaunay cell graph calculations, we used previously published tools, which can be obtained from the respective references [33,36]. The code for the neighbourhood analyses is available as part of the electronic supplementary material. This further includes the data for data I, III and IV obtained from MINS as.csv files, as well as the processes data as.json files. Data II as well as V-VIII from MINS has previously been published and is available from [22]. The processed data as.json files, codes and S1 Video can be found at: https://github.com/scfischer/fischer-et-al-2020.

## Results

### Pipeline for quantitative three-dimensional neighbourhood analyses of mouse preimplantation development

**Fig 1:**
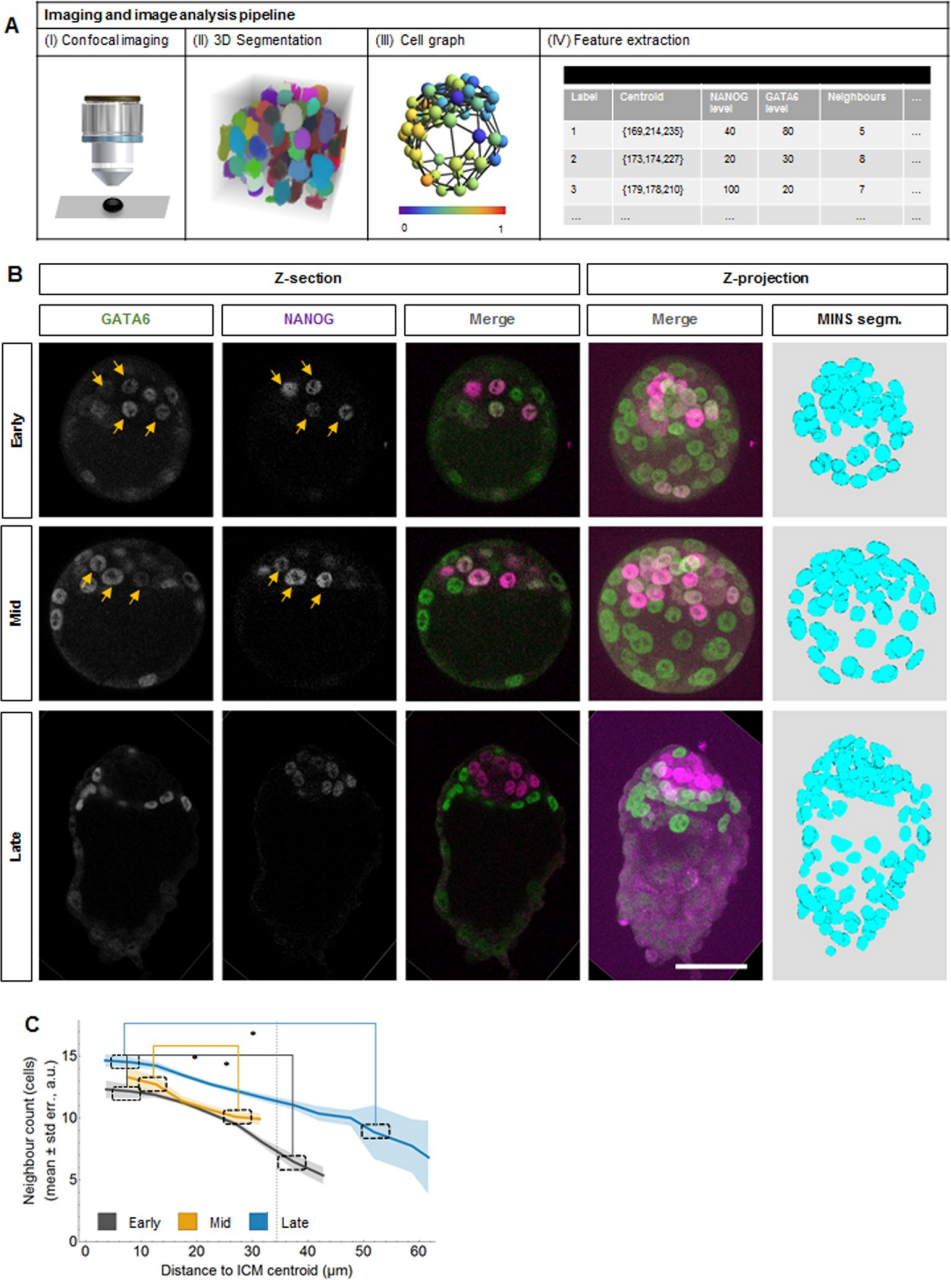
Three-dimensional imaging-based quantitative cell neighbourhood analysis of Epiblast vs Primitive Endoderm fate differentiation in preimplantation mouse embryos (data I). **(A)** Image analysis pipeline for quantitative characterization of mouse embryos. (I) Imaging with confocal laser scanning fluorescence microscope using sequential scan mode. (II) Segmentation with MINS. (III) A graphical representation of the Delaunay Cell Graph. (IV) Feature extraction for individual cells. See also S1 Fig. **(B)** Representative confocal images of mouse preimplantation embryos immunostained for GATA6 (green) and NANOG (magenta) at the indicated developmental stages. Yellow arrows point to cells co-expressing NANOG and GATA6. All embryos shown were immunostained, imaged and processed together. The first three columns are single confocal z-sections, the last columns show the maximum z-projection of the merged confocal and the segmented images. Scale bar: 50 μm. **(C)** Mean number of neighbouring cells (vertical axis) versus the distance to the centre of the ICM (horizontal axis) in early (grey), mid (yellow) and late (blue) embryos for data I. The shaded regions display the standard errors of the means. Mann-Whitney test with Bonferroni correction; *: p<0.05. Details on the number of embryos and cells analysed are in S1 and S2 Tables.

In this study, we quantitatively analyse the three-dimensional spatial distribution of cell fate markers, taking into account the single cell levels as well as the levels of the neighbouring cells (Figs 1 and S1). The quantitative immunofluorescence (QIF) analysis of NANOG and GATA6 at the single cell level in mouse preimplantation embryos at different stages of development using MINS (Modular Interactive Nuclear Segmentation) provides the cell position within the embryo, the mean expression level per nucleus and the distinction between ICM and TE (Fig 1A(I-II) and 1B, S1 Fig Step1 [33]). Our data set consists of 45 embryos from three independent experimental replicas, imaged in four confocal sessions, and staged based on the total cell number (early: 32–64, mid: 65–90, late: >90 cells; S1 and S2 Tables, data I). Due to variability in the experimental and imaging setup, we observe quantitative differences between replicas (S1 Fig Step 2). To correct this, we align the data according to NANOG and GATA6 threshold values for each experiment (see S1 Text). Based on the common thresholds, we identify four discrete cell populations: double positive (DP: N+ and G6+), double negative (DN: N- and G6-), NANOG+/GATA6- (Epi progenitor) and NANOG-/GATA6+ (PrE progenitor). The proportions of the populations in the ICM at the different developmental stages show similar trends as previously published data (S2A Fig, [22]). In particular, the proportion of DP cells decreases from early to mid to late blastocysts, and the proportion of PrE progenitor cells increases more than the proportion of Epi progenitor cells.

To investigate the three-dimensional distribution of cells, we use the Delaunay Cell Graph (DCG) to approximate the nearest neighbours of each cell (Fig 1A (III), S1 Step 3, see also S1 Text and [12,36]. The population analyses of TE cells neighbouring ICM cells and all TE cells shows that in early and mid embryos, the TE contains a large proportion of cells that is not DN and hence can provide FGF signalling to the ICM cells (S2B and S2C Fig). Therefore, for our analysis, we consider all ICM cells and those TE cells that are neighbouring at least one ICM cell (S1 Fig Step 4).

Consistent with the presence of the blastocoele, we observe a higher number of neighbours, i.e. a higher cell density, close to the ICM centroid compared to the edge of the ICM irrespective of developmental stage and cell population type, except for DN cells in early and mid blastocysts (Figs 1C and S3A). We also find that for all developmental stages, the cells of the different population types have comparable numbers of neighbours (S3B Fig). Altogether, our data processing allows us to obtain for each ICM cell and its neighbours their position, the expression levels for NANOG and GATA6, the population type and the number of neighbours, resulting in a description of the local cell neighbourhood (see Terminology Box) (Fig 1(IV)).

Similar to previous work, the population analyses further show the presence of DN cells in the ICM already in early blastocysts with proportions increasing as development progresses (S2A Fig; [4,9,20,22]). It has been proposed that these cells might correspond to Epi cells that have downregulated NANOG expression [22]. In order to assess if this cell population has any identifiable pattern of neighbour number or location within the ICM, we analysed their distribution and neighbour number (S3A and S3B Fig). While the other three cell population types have more neighbours closer to the ICM centroid, consistent with a higher cell density, we only find this pattern in the DN cell population in late embryos. This indicates that at the late stage, the DN cells could indeed be Epi cells that have downregulated NANOG. In early embryos, there is no clear pattern and the cell density around DN is comparable at any position within the ICM at this stage. Further studies will be needed to characterize the nature, origin and fate of these DN cells.

In summary, we integrate QIF measurements with an approximation for the nearest neighbours of a cell to obtain a data set that enables studying three-dimensional local cell neighbourhoods in the ICM of early, mid and late mouse blastocysts.

### Clustering of population types is observed in the ICM of early embryos

**Fig 2:**
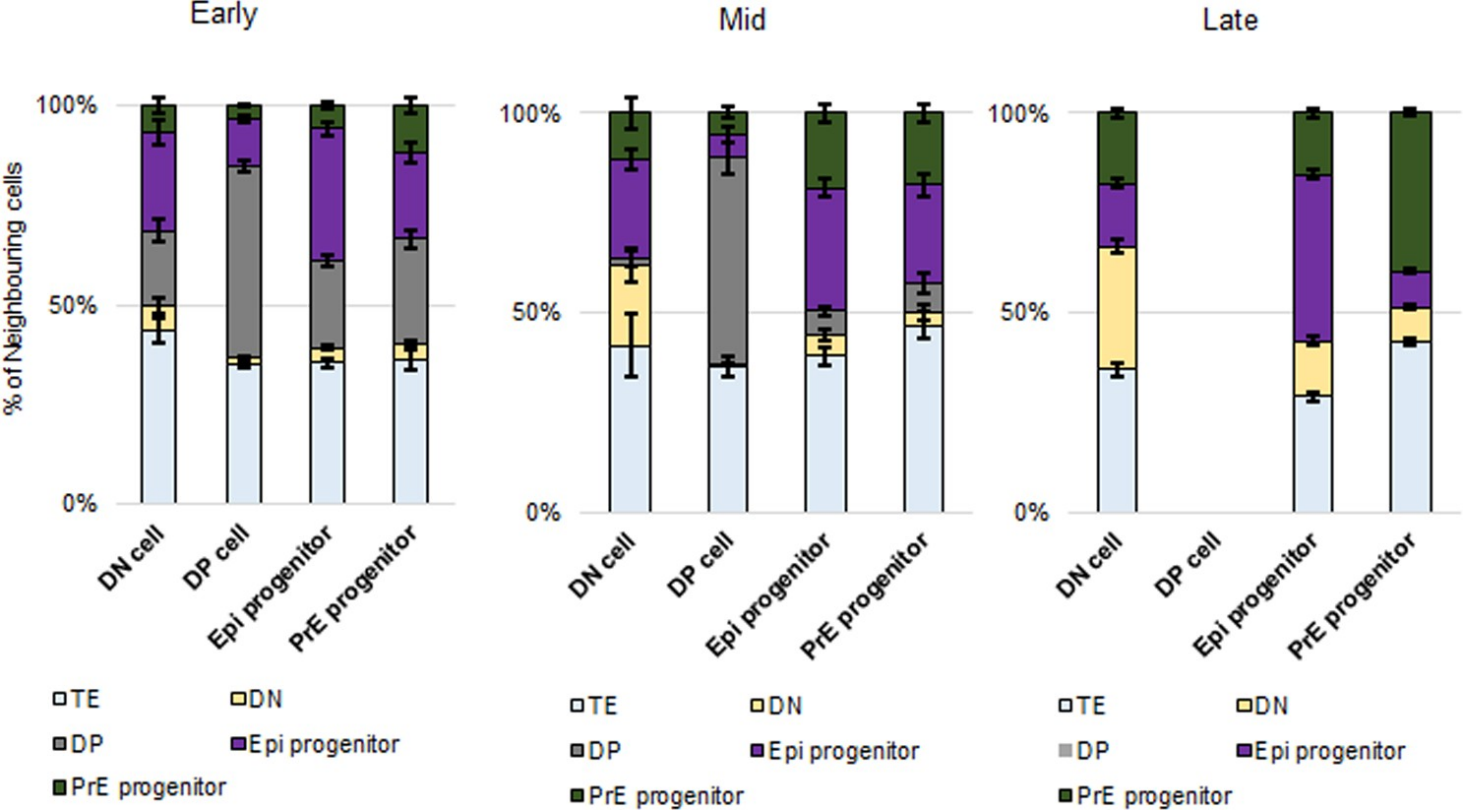
Neighbour composition analysis indicates clustering of Epi progenitor cells and DP cells (data I). The proportion of neighbouring cells of one of the five populations TE, DN, DP, Epi progenitor and PrE progenitor are displayed for DN, DP, Epi progenitor and PrE progenitor cells in in early (left panel), mid (centre) and late (right) embryos for data I. Error bars indicate the standard errors of the means. See S4 Fig for the statistical z-test results. Details on the number of embryos and cells analysed are in S1 and S2 Tables.

We have recently shown that ICM organoids and mid/late embryos show local clustering of cells with the same population type [12]. Here, we extend this analysis to include early embryos (Figs 2 and S4 for statistical analyses). In the early blastocysts, around 40% of the neighbouring cells of an ICM cell are TE. Surprisingly, we already observe a pattern: the ICM neighbours of DP cells are mostly DP cells and the ICM neighbours of Epi progenitor are either TE or mostly Epi progenitor cells. In mid embryos, the clustering of DP cells and Epi progenitor cells remains, as previously observed [12]. For late blastocysts, we observe the expected pattern. Epi cells have a large proportion of Epi neighbours and the lowest proportion of TE neighbours, as they occupy the internal positions. PrE cells have a larger proportion of TE neighbours and a large proportion of PrE neighbours. This is consistent with PrE cells forming an epithelium between the blastocoele and the Epi cells at this late stage with some of them already migrating (see late embryo in Fig 1B, and [37]). Finally, the proportion of PrE neighbours increases for all four populations from early to mid blastocysts, consistent with the increase of PrE cells in the population distribution of the ICM (S2A Fig).

In summary, our results indicate population clustering of DP and Epi progenitor cells, which is already present in early embryos.

### DP cells in early blastocysts exhibit GATA6 expression level clusters

**Fig 3:**
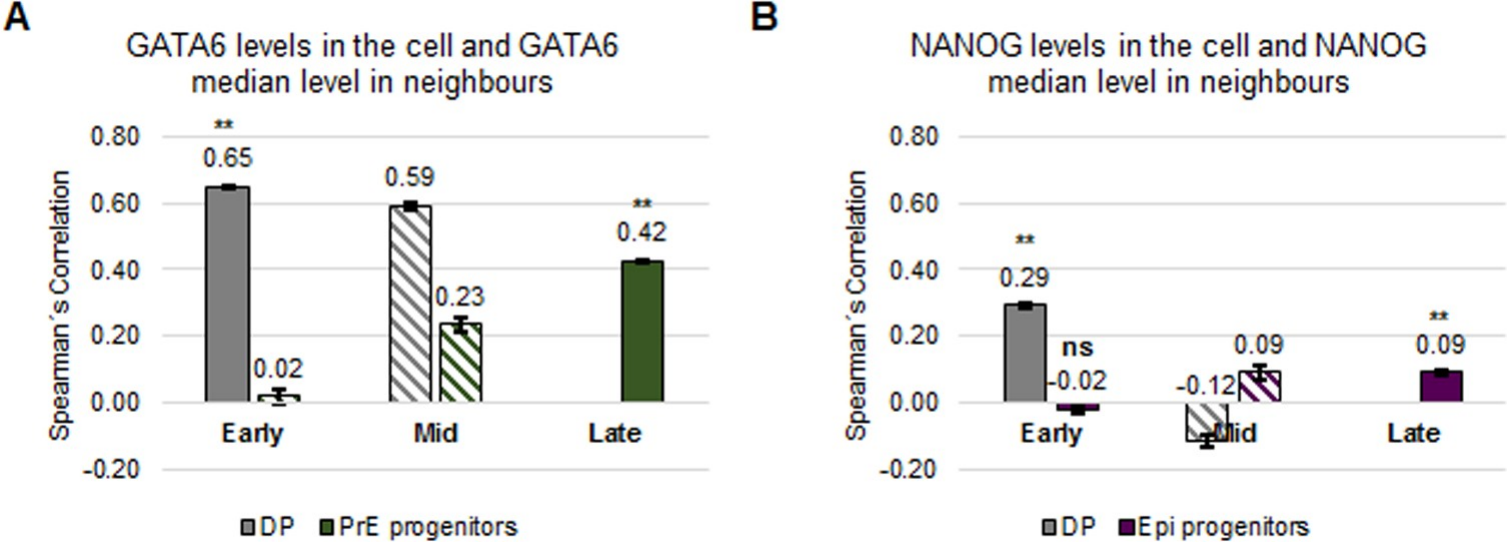
DP cells in early blastocysts cluster together according to their GATA6 levels (data I). **(A-B)** Spearman’s correlation coefficients of GATA6 levels of a cell and median GATA6 levels of its neighbours (A) or of NANOG levels of a cell and median NANOG levels of its neighbours (B) in the indicated populations in the ICM at the indicated embryonic developmental stages for data I. The error bars represent the standard errors calculated by bootstrap sampling the experimental data 100 times. **: p<0.01; Mann-Whitney test with Bonferroni correction for the null model; ns: not significant. See also S5 Fig and S1 Text for further details. Striped boxes indicate those populations composed of less than 108 cells. In those cases, no statistical analysis was performed. Details on the number of embryos and cells analysed are in S1 and S2 Tables.

Our analyses show a novel population clustering of DP and Epi progenitor cells in early blastocysts. To investigate this further, we step back from the discrete categorisation of the cells and their neighbours into population types based on high or low expression of NANOG and GATA6. Instead, we consider NANOG and GATA6 expression levels as continuous parameters. This approach allows us to measure the correlation strength of the levels of NANOG or GATA6 in a cell with the NANOG or GATA6 levels of its neighbours, respectively. We chose to use Spearman’s correlation coefficient as it requires less assumptions, i.e. it does not require bivariate normal data and it measures monotonic, not just linear relationships like the Pearson´s correlation coefficient does (Fig 3 and S5 Fig, [35], see Material and Methods for the classification of correlation strengths, and also S1 Text for further details on the analysis).

Given the dependence of the validity of a correlation analysis on cell number and the typically low number of cells present in mouse preimplantation embryos, we perform a correlation sensitivity analysis (S1 Text). This sensitivity analysis shows that more than 108 cells per cell population are required to obtain robust results with less than 3% variability. For completeness, we included all results in the following plots. However, those that are obtained from less than 108 cells are marked with stripes and should not be relied upon.

The correlation analysis of the GATA6 level in a cell and the median GATA6 level of its neighbours indicates strong positive correlations in DP cells in early and moderate in PrE progenitor cells in late blastocysts (Fig 3A and S5C Fig). The neighbourhood correlation analyses of NANOG result in no correlation, very weak or weak correlations for all populations in early and late blastocysts (Fig 3B and S5D Fig).

To ensure that the positive correlation results do not arise randomly or are affected by specific constraints on NANOG/GATA6 distributions, we also investigated whether the correlation values are significantly different from those of a null model (see S1 Text for further details about the null models tested and used). We find that the neighbourhood correlations in the null model are significantly lower than those found for DP cells in early blastocysts and Epi progenitor as well as PrE progenitor cells in late blastocysts. Hence, the correlation values observed for GATA6 do not arise randomly. This is the first time that local correlations of GATA6 levels have been documented for early blastocysts.

It has been proposed that Epi fate reinforces PrE fate in neighbouring cells via FGF4 [4,6–8,11]. This hypothesis would predict a positive correlation of NANOG levels of a cell with GATA6 levels in its neighbouring cells and GATA6 levels of a cell and NANOG levels in its neighbours. However, this hypothesis cannot be tested in this data set as all cell populations in mid blastocysts contain less than 108 cells (S2 Table, S5 Fig; see below and S8C and S8D Fig for more on this issue).

These results suggest that DP cells in the ICM form GATA6 expression level clusters. This local distribution of DP is first present in the early blastocysts. Furthermore, NANOG expressing cells do not cluster according to their expression levels.

### NANOG expression levels correlate with the number of neighbours in early blastocysts

**Fig 4:**
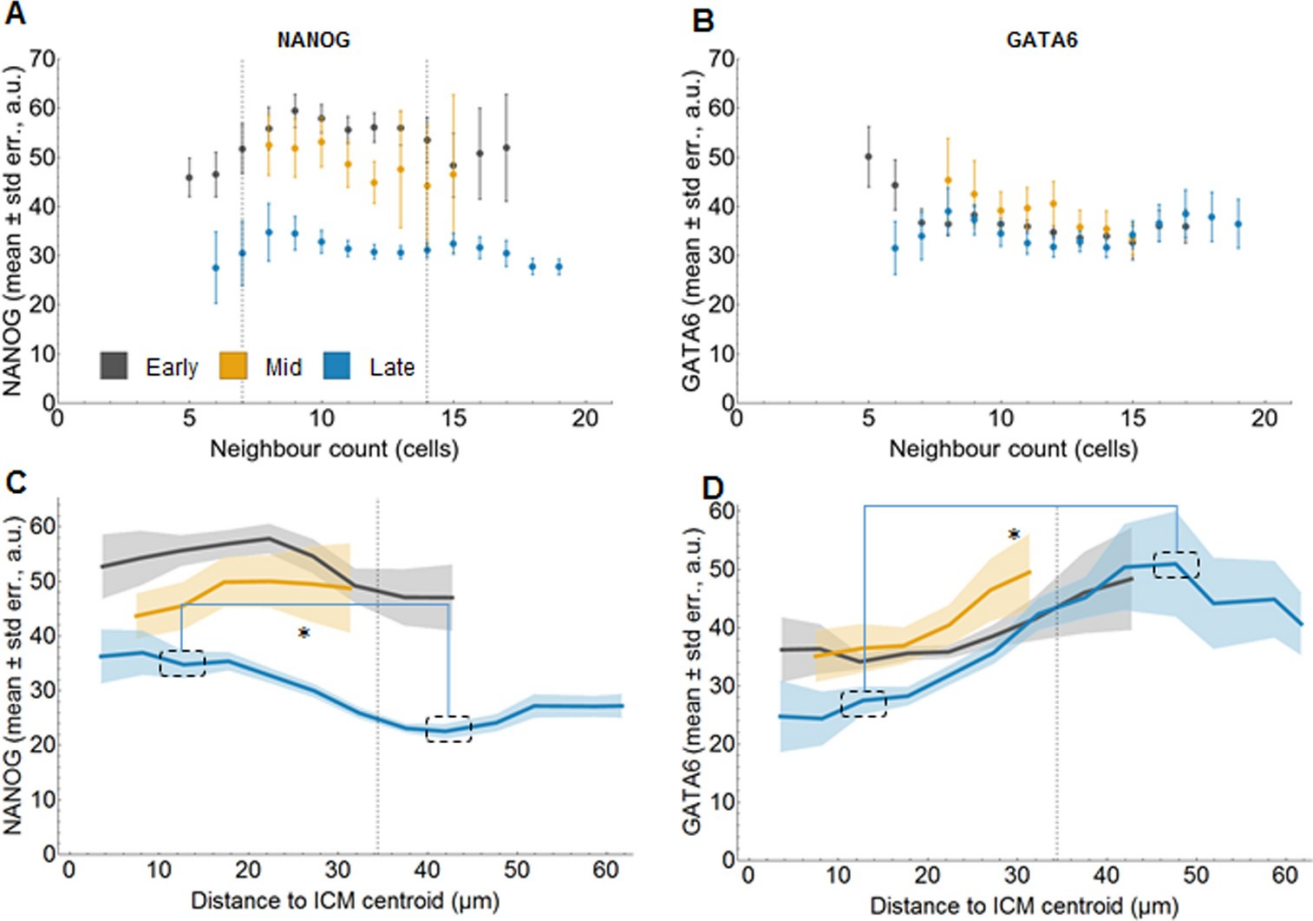
Cells with nine neighbours express highest NANOG levels in early and mid blastocysts (data I). **(A-B)** Mean level of NANOG (A) or GATA6 (B) (vertical axis) versus the number of neighbours (horizontal axis) for ICM cells in early (grey), mid (yellow) and late (blue) blastocysts for data I. The error bars display the standard errors of the means. See also S7A Fig. **(C-D)** Mean level of NANOG (C) or GATA6 (D) (vertical axis) versus the distance to the ICM centroid (horizontal axis, binned in 5 μm groups) for ICM cells in in early (grey), mid (yellow) and late (blue) blastocysts for data I. Mann-Whitney test with Bonferroni correction; *: p<0.05. See also S7B Fig. For simplicity, only selected significant results are indicated for NANOG levels in late embryos, for full statistical results see S7C Fig. Shaded regions display the standard errors of the means. Details on the number of embryos and cells analysed are in S1 and S2 Tables.

We next test whether Epi progenitor clustering is related to positional information within the ICM. To do this, we tested whether there are local or global positional features related to NANOG and GATA6 expression levels (Fig 4).

To investigate the local positional features, we analysed the relation between expression levels and total number of neighbours (Fig 4A and 4B). We observe, particularly in early blastocysts, a peak in NANOG expression in cells with 7 to 14 neighbours, with the maximal level in cells with 9 neighbours (Fig 4A). Three-dimensional illustrations of the number of neighbours and the NANOG levels in the ICMs of the individual early blastocysts support this finding (S6 Fig). Performing the same analyses for GATA6 expression levels, we detected no clear relation at any stage (Fig 4B).

The number of neighbours favouring higher NANOG expression levels in a cell might be an artefact of our DCG approach. To assess this, we performed a sensitivity analysis of the DCG (see S1 Text for further details). It demonstrates that the DCG does not favour a 9 neighbour topology for the ICMs. In early embryos, the DCG favours 2–4 neighbours, which is lower than what we observe in our results for the NANOG analysis.

To investigate further the robustness of our results, we analysed whether there was a specific effect from the particular geometry of the embryos. Therefore, we compared the results for expression level versus number of neighbours with those for the null model, introduced for the correlation analysis (S7A Fig, and see S1 Text for further details). We find that in the null model, the expression levels of NANOG or GATA6 do not correlate with the number of neighbouring cells at any stage.

For the global positional features, we investigate the relation between expression levels and the position of a cell relative to the centroid of the ICM (Fig 4C and 4D, S7B Fig for the null model results, S7C for the statistical analysis of the experimental data, and Materials and Methods). Only in late blastocysts, we observe statistically significant higher NANOG levels closer to the centroid and lower levels away from it (Fig 4C and S7C Fig). This is consistent with Epi cells being located at the centre. The equivalent analysis for GATA6 expressing cells also shows a global positional effect: the highest GATA6 expressing cells are located distally from the ICM centroid in late blastocysts, consistent with their final position facing the blastocoele (Fig 4D and S7C Fig).

In summary, these results show that there is a clear interrelation between the number of neighbouring cells and NANOG expression levels in early blastocysts. Conversely, the number of neighbours does not correlate with GATA6 expression levels. A global pattern is only present in the ICM of late embryos, likely coincident with the resolution of the sorting process.

### GATA6 level clusters arise in DP and PrE progenitor cells in early and mid blastocysts

In the next step, we extend our analysis to a larger data set ([22], S1 and S2 Tables, data II). This allows us to ensure the robustness of our observation. Furthermore, we are able to extend the correlation analysis to the populations for which our initial data (data I) did not include enough cells. The larger data set was generated in a different laboratory with a slightly different experimental set up (see Material and Methods for details). Factors like the variability of the data or the presence of outliers affect the value of a correlation coefficient ([35,38], see also Sup. Info for further details). Therefore, we expect qualitative but not quantitative agreement between the results for data I and data II.

**Fig 5:**
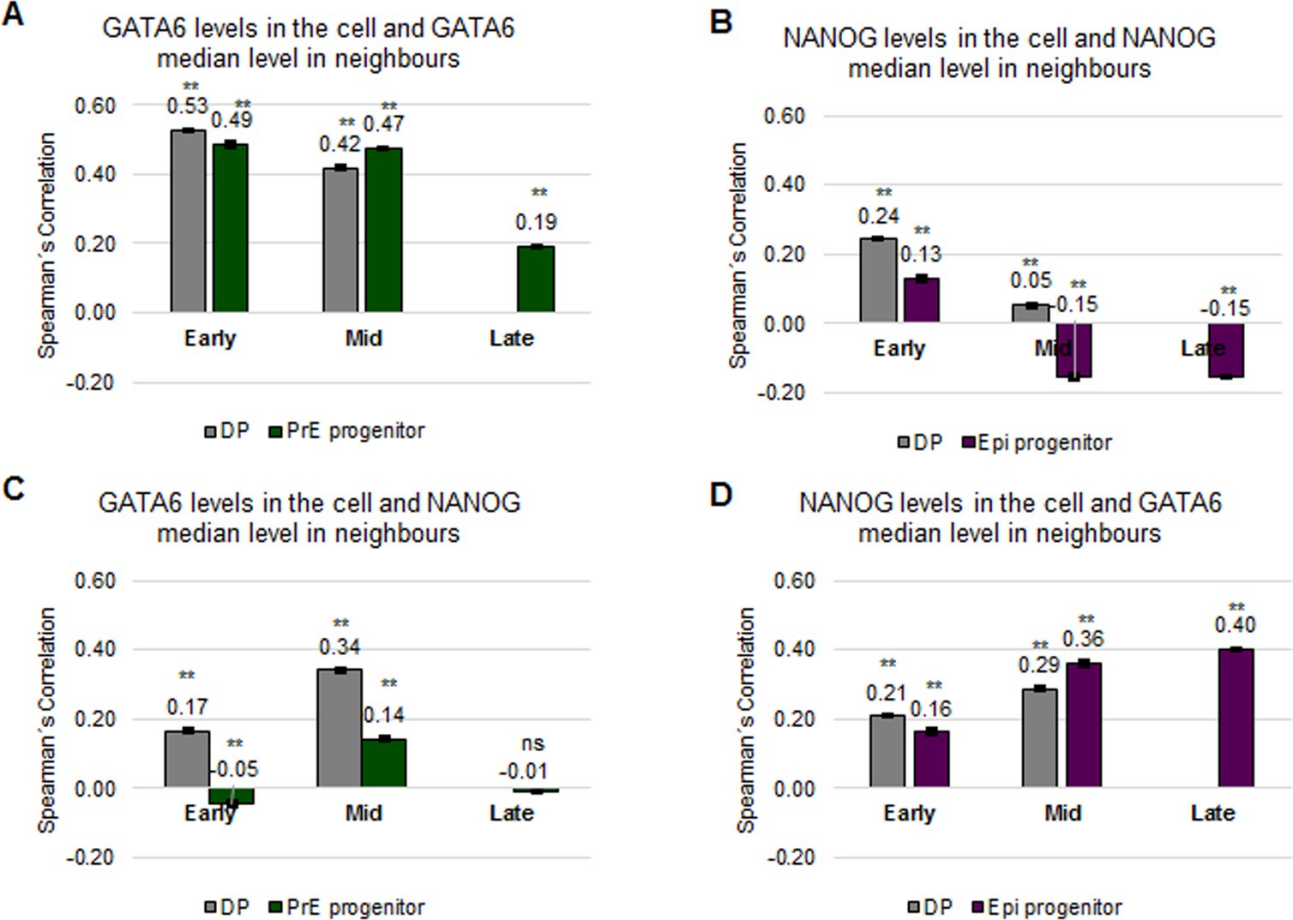
DP as well as PrE progenitor cells in early and blastocysts cluster together according to their GATA6 levels (data II). **(A)** Spearman’s correlation coefficients of GATA6 levels of a cell and the median GATA6 levels of its neighbours in the indicated populations in the ICM and embryonic developmental stages (). **: p<0.01 Mann-Whitney test with Bonferroni correction for comparison with the null model. The error bars represent the standard errors calculated by bootstrap sampling the experimental data 100 times, here and in B, C and D. See S1 Text for further details. **(B)** Spearman’s correlation coefficients of NANOG levels of a cell and the median NANOG levels of its neighbours in the indicated populations in the ICM and embryonic developmental stages. **(C)** Spearman’s correlation coefficients of GATA6 levels of a cell and the median NANOG levels of its neighbours in the indicated populations in the ICM and developmental stages. **(D)** Spearman’s correlation coefficients of NANOG levels of a cell and the median GATA6 levels of its neighbours in the indicated populations in the ICM and embryonic developmental stages. Details on the number of embryos and cells analysed are in S1 and S2 Tables.

We find that the main conclusions from our previous observations also hold in this second data set: in early blastocysts, we obtain GATA6 expression level clusters in DP cells (Fig 5A) and NANOG levels are highest in cells with nine neighbours (S8A Fig). NANOG expression level clusters and a correlation between number of neighbours and GATA6 levels was not observed (Fig 5B and S8B Fig). Interestingly, in this second data set, we further find moderate positive correlations for GATA6 in DP cells in mid blastocysts as well as PrE progenitor cells in early and mid blastocysts. These correlations do not arise randomly (Fig 5A, comparison with null model) nor are they an artefact of inter-embryo variability (S8C–S8F Fig).

In addition, we could test the hypothesis of Epi fate reinforcing PrE fate in neighbouring cells [4,6–8,11]. The correlation between GATA6 levels in a cell and NANOG levels in its neighbours is very weak (or weak in the DP cells, S5C Fig) and between NANOG levels in a cell and GATA6 in its neighbours is weak (Fig 5D) or moderate (S5B Fig). Hence Epi fate reinforcing PrE fate in neighbouring cells is still an outstanding question. These results indicate that even if Epi cells can promote PrE fate in the neighbours, the mechanism does not rely on a direct translation of the levels of NANOG in a cell to the GATA6 levels expressed in its neighbours. Furthermore, we observe a global pattern for NANOG expression in mid and late embryos (S8G Fig). In mid embryos, cells at the edge of the ICM have the highest expression of NANOG. In late embryos, we get a pattern with NANOG expression highest in the centre of the ICM and GATA6 expression highest in cells at the edge of the ICM (S8G and S8H Fig and S9 Fig for statistical analysis). Taken together with the results from data I (Fig 4), we conclude that a clear global pattern starts to arise in mid blastocysts.

Altogether, our results reveal a novel three-dimensional pattern in the distribution of the population types in ICM cells in early blastocysts. Local positional features and local expression level features characterize this pattern (Figs 3–5 and S5–S9 Figs).

### Two simple rules can generate the population composition observed in early blastocysts

**Fig 6:**
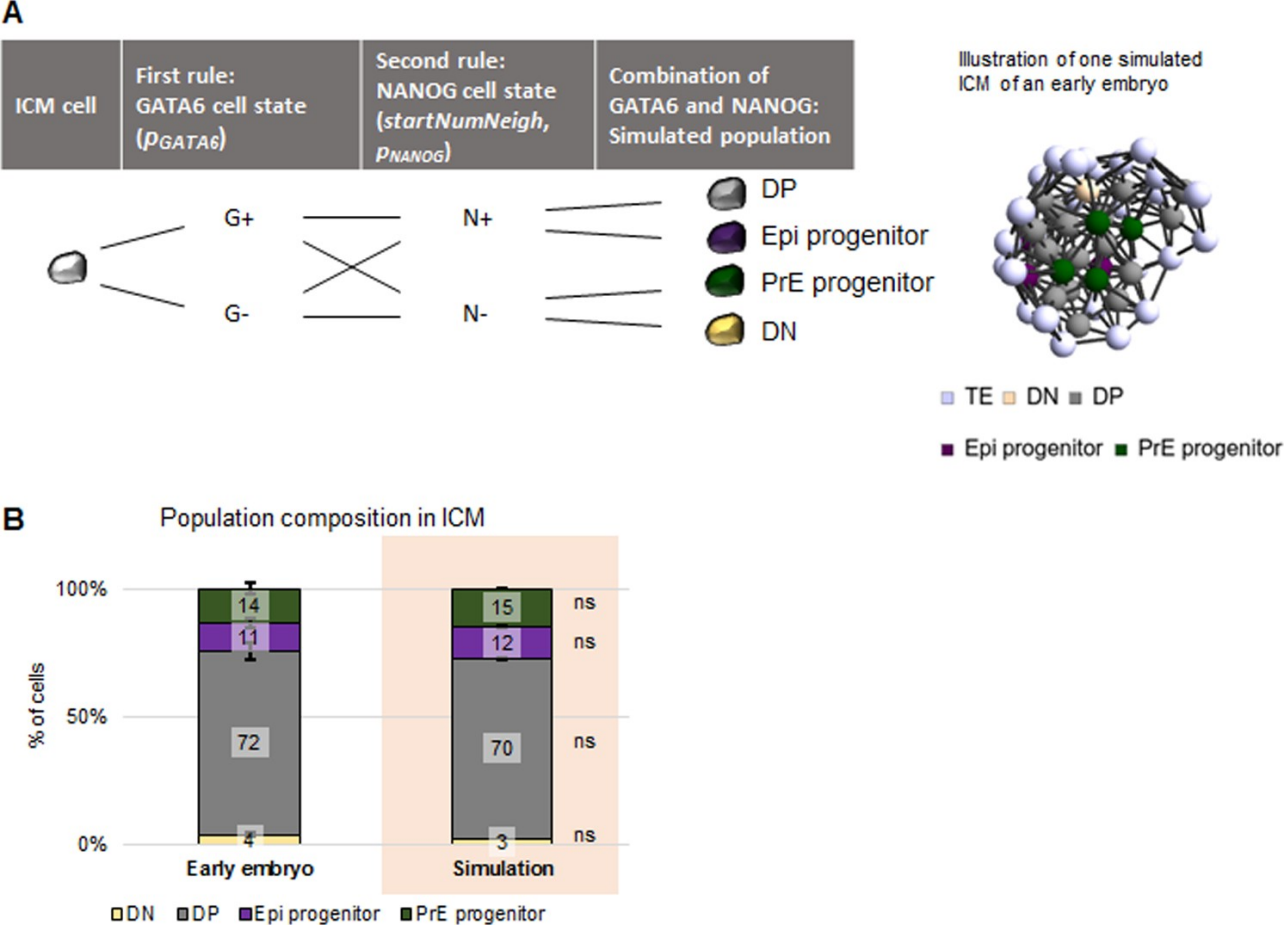
Simulation of two rules recapitulates population composition in early blastocysts. **(A)** Diagram depicting the population type assignment to the cells in the simulations (left) and an example of a simulated three-dimensional cell distribution (right). See main text and S1 Text for further details on the two rules and parameters. **(B)** Proportion of DN, DP, Epi progenitor and PrE progenitor ICM cells in early blastocysts in data II (first bar) and upon simulating the model (pink background) presented in (A). For the parameters used, see the main text and S1 Text. z-test; *: p<0.05. The error bars indicate the standard errors of the means.

Our results indicate that the complex three-dimensional distribution of the population types can be broken down into GATA6 level clustering and NANOG level dependence on number of neighbours. To test this, we implemented a computer simulation based on these two rules and compared the results to the population composition of the experimental data. Different to traditional rule-based models [39], we do not aim at modelling cellular mechanisms. Our approach aims at validating the simple rules that we identified to describe the population type pattern (Fig 6, [12]).

As geometrical basis for the simulations, we used the measured cell centroid positions. To obtain the four populations, we assigned each cell a G6+ or G6- and N+ or N- cell state (see Terminology Box), respectively, according to the following two rules with three parameters (Fig 6A):

1. G6+ cells are clustered according to their GATA6 levels; in the model, this is achieved by randomly assigning the cell state G6+ to a cell with a probability of 85% (*p*_*GATA*6_ = 85%), otherwise the cell is G6-;
2. N+ cell state correlates with the number of neighbours; in the model, this is achieved by setting N+ cells as those with nine or close to nine neighbours (*startNumNeigh* = 9) up to 82% (*p_NANOG_* = 82%), otherwise the cell is N-.

Hence, we input expression levels for GATA6 and NANOG separately and as output we obtain the four populations as combinations of the two expression levels.

The values for all three parameters are obtained from the experimental data II [22]. The parameters *p*_*GATA*6_ and *p_NANOG_* are the proportions of ICM cells positive for GATA6 or NANOG expression, respectively. Hence, *p*_*GATA*6_ is the proportion of DP and PrE progenitor cells and *p_NANOG_* is the proportion of DP and Epi progenitor cells. Combining this information and rules for each cell, we determine its simulated population type. The results of the simulations for these parameter values are comparable to the experimental data, indicating that implementing these two rules allows the generation of the embryos with the observed population composition in early blastocysts (Fig 6B).

To assess the robustness of the model, we perform a parameter sensitivity analysis (see S1 Text). This analysis shows that the simulation results are very robust with respect to the starting number of neighbours. Hence, within the observed range, cell density is not determinant for the proportion of cell fate allocation. The sensitivity analysis further shows that the model is sensitive to changes in the proportion of G6+ and N+ cells. Altogether, this indicates that the main parameter affecting the cell population composition in early embryos is the proportion of cells in a particular cell state.

In summary, these results show that two simple rules for assigning cell state are capable of representing, to a very good approximation, the population composition observed in the ICM of early blastocysts.

### NANOG promotes GATA6 expression level clusters

**Fig 7:**
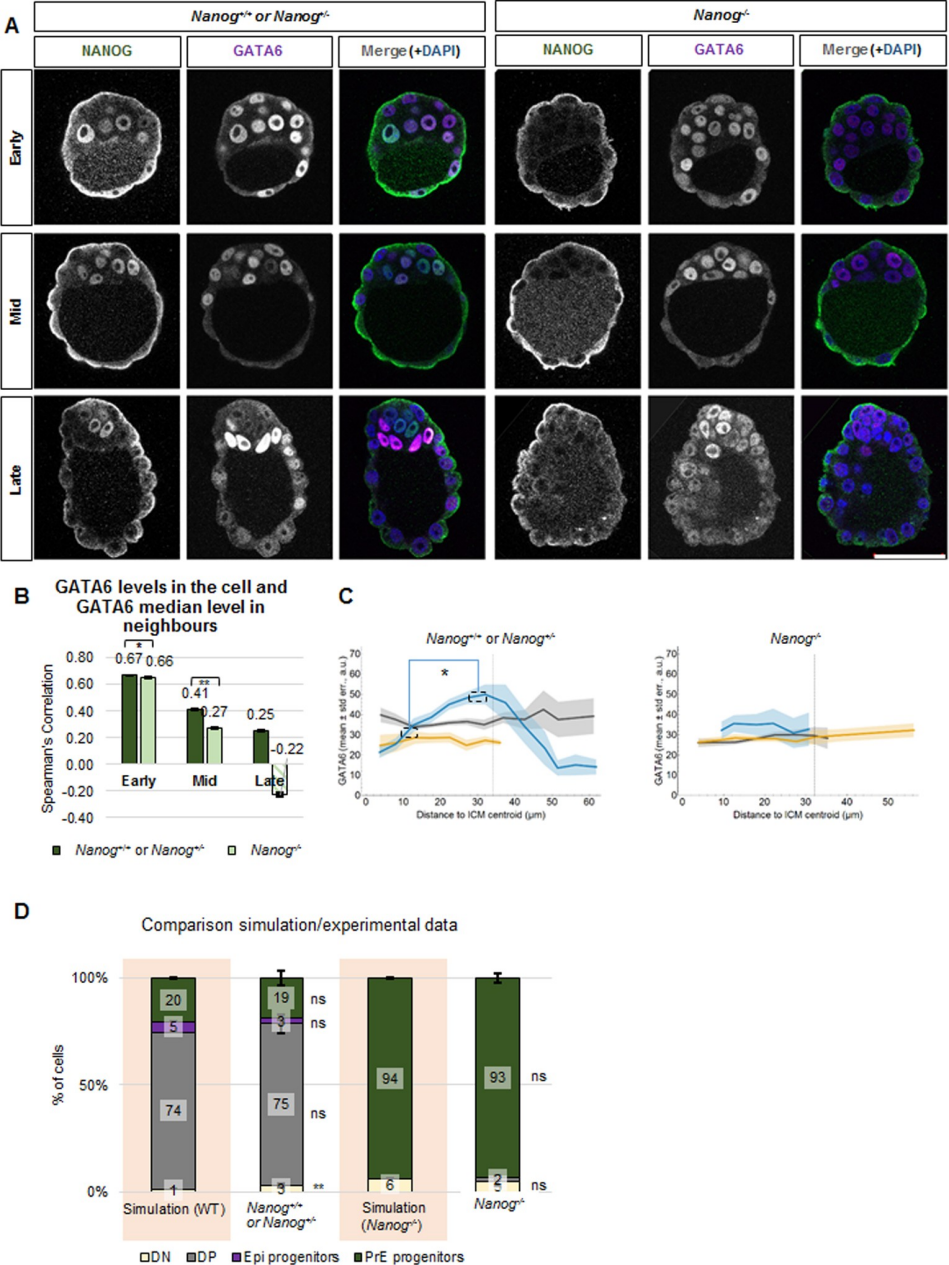
NANOG regulates GATA6 neighbourhood patterning (data III-IV). **(A)** Representative confocal z-sections of *Nanog*^*+/+*^ or *Nanog*^*+/-*^ (left) and *Nanog*^*-/-*^ (right) embryos immunostained for GATA6 (green) and NANOG (magenta) at the indicated developmental stages. Embryos from the same developmental stage were immunostained, imaged and processed together. Scale bar: 50 μm. **(B)** Spearman’s correlation coefficients of the GATA6 level of a cell and the median GATA6 level of its neighbours in G+ populations (DP and PrE progenitor) in the ICM at different embryonic developmental stages in *Nanog*^*+/+*^ or *Nanog*^*+/-*^ and *Nanog*^*-/-*^ embryos for data III and IV. Mann-Whitney test with Bonferroni correction between genotypes; *: p<0.0.5. The Mann-Whitney test with Bonferroni correction for the comparison with the null model results in statistically significant differences in all cases (**: p<0.01; not shown in the figure). The error bars represent the standard errors calculated by bootstrap sampling the experimental data 100 times. Note that in some cases they are so small that they cannot be appreciated in the figure. DP and PrE progenitor cells in the *Nanog*^*+/+*^ or *Nanog*^*+/-*^ were pooled together to simplify the analysis and to increase the total analysed cell number. Striped boxes indicate those populations composed of less than 108 cells. In those cases, no statistical analysis was performed. Details on the number of embryos and cells analysed are in S1 and S2 Tables. **(C)** Mean level of GATA6 (vertical axis) versus the distance to ICM centroid (horizontal axis) for ICM cells in *Nanog*^*+/+*^ or *Nanog*^*+/-*^ (left) and *Nanog*^*-/-*^ (right) early (grey), mid (yellow) and late (blue) blastocysts for data III and IV. Mann-Whitney test with Bonferroni correction; *: p<0.05. For simplicity, only selected significant results are indicated, for GATA6 expression levels. For full statistical results, see S9 Fig. Shaded regions indicate the standard errors of the means. Details on the number of embryos and cells analysed are in S1 and S2 Tables. **(D)** Proportion of simulated DN, DP, Epi progenitor and PrE progenitor ICM cells in wild-type or *Nanog*^*-/-*^ early blastocysts (left, pink background) and proportions obtained in the experimental data (right, data III and IV). Error bars indicate the standard errors of the mean. z-test; **: p<0.01. Note: the DP population present in the *Nanog*^*-/-*^ mutant embryos corresponds to five cells, which lie above the calculated threshold. Details on the number of embryos and cells analysed are in S1 and S2 Tables.

Previous studies indicate that NANOG represses GATA6 during Epi versus PrE differentiation [4]. To analyse if NANOG also regulates GATA6 neighbourhood features, we performed our neighbourhood analyses in *Nanog* mutant embryos (Fig 7).

We analyse and compare *Nanog*^*+/+*^ or *Nanog*^*+/-*^ (73 embryos; S1 Table, data III) with *Nanog*^*-/-*^ (19 embryos; S2 Table, data IV) results. In this case, we pool together *Nanog*^*+/+*^ and *Nanog*^*+/-*^ as there is no dosage effect [4,40]. As previously shown, early and mid *Nanog* mutant blastocysts do not show any phenotypic defects until late stages (Fig 7A; [4,7]). The detailed single cell quantitative analysis of GATA6 expression in the absence of *Nanog* shows decreased values in mid and late embryos (S1A Fig), consistent with previous reports indicating PrE specification defects in the mutants [4,7,41].

We start by analysing the clustering of cells according to their GATA6 expression levels (Fig 7B). In the absence of *Nanog*, we still observe clusters of GATA6 expressing cells, reflected in the strong correlation found in GATA6 levels in a cell and median levels in the neighbours in early embryos. However, in the absence of *Nanog*, the correlation decreases from moderate to weak in mid blastocysts. Finally, in the late mutant blastocysts, there are fewer than 108 N-G6+ cells, hence the observed weak anti-correlation cannot be relied upon (see S1 Text). This low cell number probably results from the apoptosis of ICM cells at this stage in *Nanog* mutants [7].

We next analyse the global positional feature (see Terminology Box; Fig 7C). This analysis shows that the distribution of GATA6 expressing cells is altered in the absence of *Nanog* (Fig 7C and S10B Fig for statistical analysis): the clear distribution of highest GATA6 expressing cells located away from the ICM centroid due to cell sorting disappears and cells express similar GATA6 levels independently of their position in late blastocysts.

Finally, we were interested in testing whether our simulations can generate the population composition in *Nanog* mutants (Fig 7D). To simulate the wild-type situation, we use data from early *Nanog*^*+/+*^ or *Nanog*^*+/-*^ embryos (44 embryos; see Material and Methods). In the *Nanog* mutant simulations, we set the proportion of N+ cells to 0. We did not detect any statistically significant differences between simulations and experimental results both in *Nanog*^*+/+*^ or *Nanog*^*+/-*^ and *Nanog*^*-/-*^ embryos. Hence, our model is also capable of reproducing the mutant phenotype.

Note: we also had access to quantitative single cell data from a previously published data set composed of 19 *Gata6*^*+/+*^, 28 *Gata6*^*+/-*^, and 15 *Gata6*^*-/-*^ embryos (5). However, we decided not to perform an analysis, since in most cases, the number of cells per population type was below 108 cells.

Altogether, these results suggest NANOG is involved in the neighbourhood regulation of GATA6 expressing cells, coordinating GATA6 expression levels to form the observed clusters and their global position in late embryos.

### FGF/ERK signalling promotes GATA6 expression level clusters and inhibits NANOG expression level clusters

**Fig 8:**
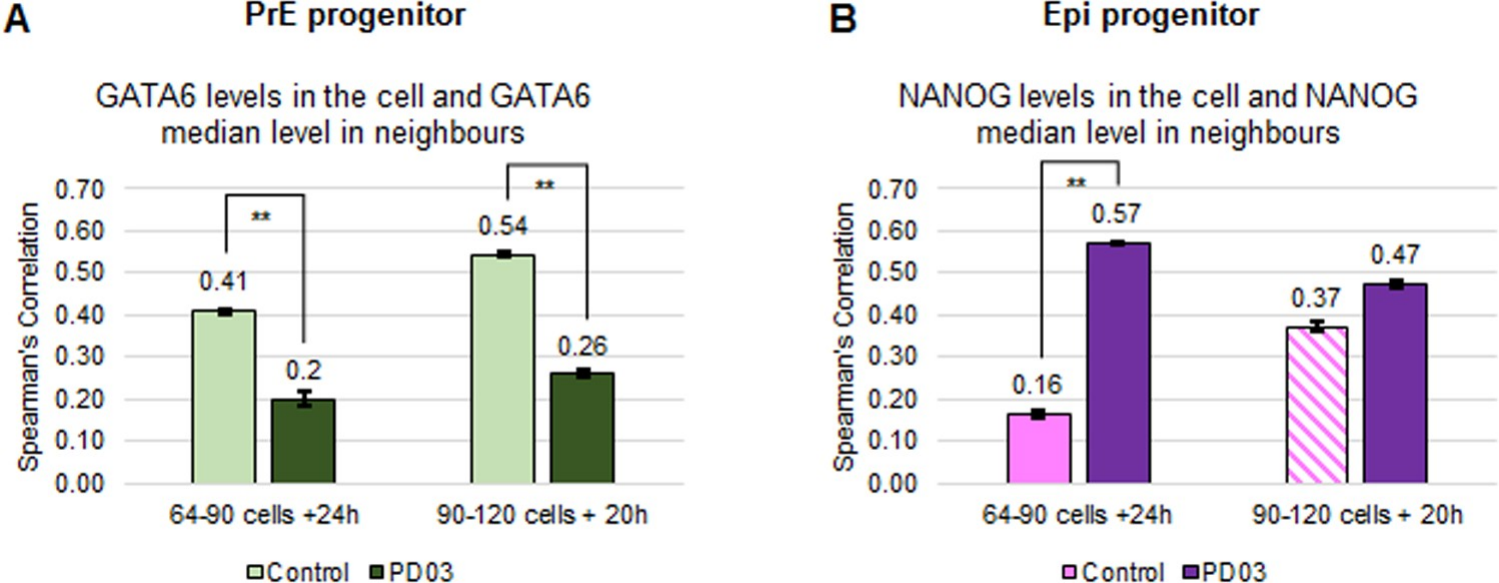
Inhibition of FGF/MAPK signalling inhibits GATA6 clusters and promotes NANOG clusters (data V-VIII). **(A-B)** Spearman’s correlation coefficients of GATA6 levels of a cell and the median GATA6 levels of its neighbours in PrE progenitor cells (A) or NANOG levels of a cell and the median levels of its neighbours in Epi progenitor cells (B) in control (light green or magenta, respectively; data V and VI) and PD03 treated embryos for 24 h or 20 h (dark green or magenta, respectively; data VII and VIII). The error bars represent the standard errors calculated by bootstrap sampling the experimental data 100 times The Mann-Whitney test with Bonferroni correction for the comparison with the null model results in statistically significant differences in all cases (**: p<0.01; not shown in the figure). Striped boxes indicate those populations composed of less than 108 cells. In those cases, no statistical analysis was performed. Details on the number of embryos and cells analysed are in S1 and S2 Tables 1. See also S11 and S12 Figs.

As NANOG and GATA6 expression is affected by FGF/ERK signalling (reviewed in [42]), we next investigated if this signalling pathway is involved in the regulation of the local three-dimensional cell neighbourhood features (see Terminology Box). We used the available data sets (Fig 8; [22]; S1 and S2 Tables, data V-VIII). We focused our three-dimensional analyses on mid blastocysts treated for 24 h or 20 h with PD03, an inhibitor of FGF/ERK signalling. This regime promotes NANOG upregulation, without completely abolishing GATA6 expression (S1A and S1B Fig). The data set also includes the use of an FGFRi, however in most cases, the number of cells per population type was below 108 cells. Hence, an analysis of this data would not yield reliable results. The data set further includes FGF4 treatments, which result in almost entirely PrE progenitor cells and therefore do not allow investigating the effect on NANOG or GATA6 neighbourhood features.

We first analyse the effect of the decreased FGF/ERK signalling on GATA6 expression level clustering. This shows decreased GATA6 correlation between PrE progenitor cells and their neighbours (Fig 8A). These results indicate that active FGF/ERK signalling is required to coordinate GATA6 expression levels between neighbouring cells to form the clusters. Concomitantly, we also observe an increased NANOG correlation between Epi progenitor cells and their neighbours, which reflects a NANOG expression level clustering for this population (Fig 8B).

The analysis of the local positional features (see Terminology Box) gives inconclusive results, as the control-cultured embryos did not show the clear pattern observed in the freshly flushed embryos (S12A and S12B Fig). The same applies for the global positional features related to GATA6 expression levels and cell position within the ICM (S12C and S12D Fig). Regarding global positional features related to the NANOG expression levels, there are no differences between control and treated embryos, albeit the absolute levels: highest NANOG expressing cells are closest to the centroid, consistent with these embryos being in late stages (S12E and S12F Fig).

These results, together with previously published results, are consistent with a scenario in which active FGF/ERK signalling is required for regulating NANOG expression in neighbouring cells, and for generating GATA6 expression level clusters.

## Discussion

**Fig 9:**
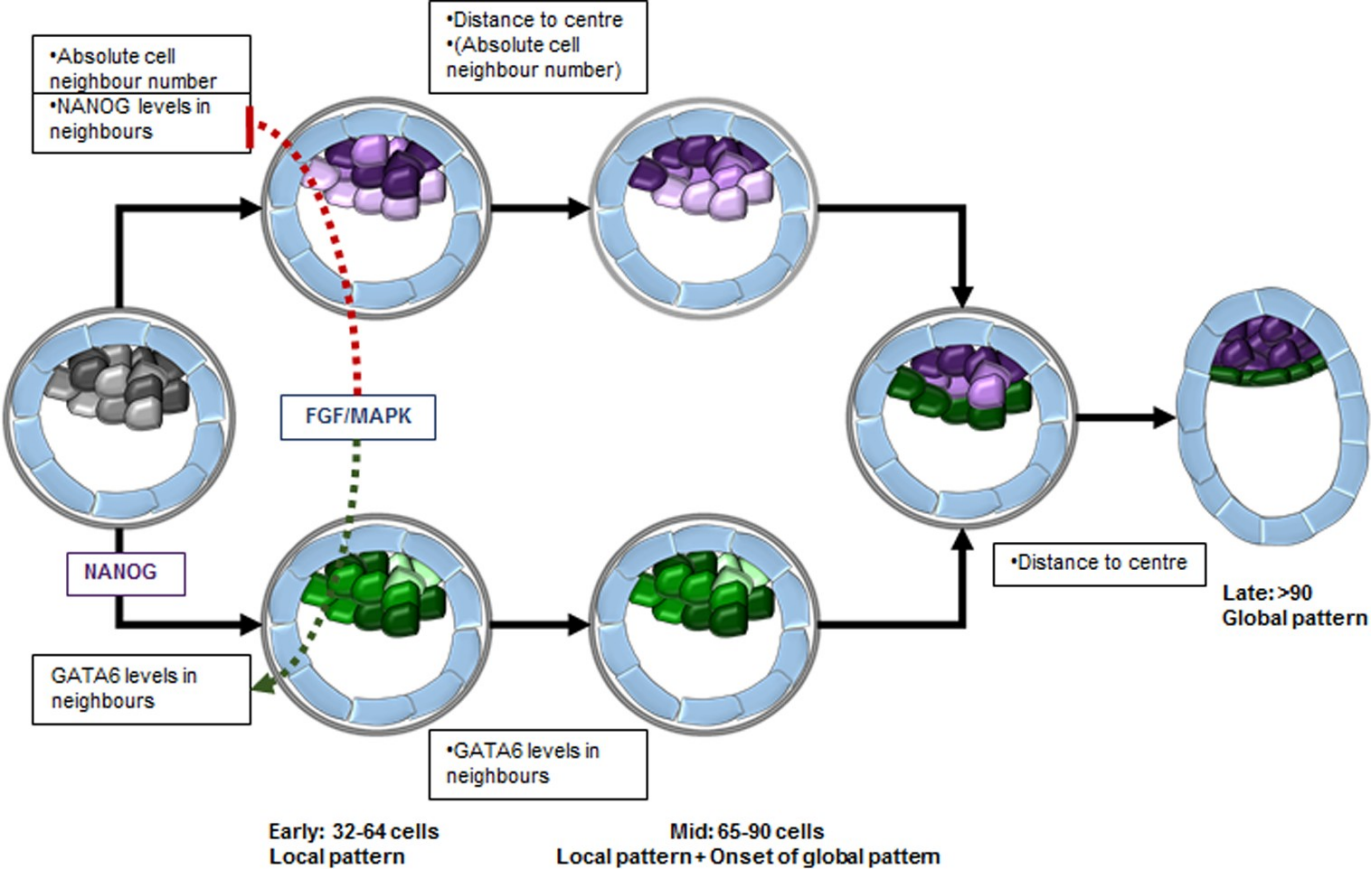
Model for transition from local to global patterns during cell fate decision in the ICM from early to late blastocysts. In early blastocysts, the majority of the ICM cells co-express NANOG and GATA6 but at different levels following a three-dimensional local pattern. NANOG levels in a cell correlate with the absolute cell neighbour number: cells with nine neighbours express the highest NANOG levels. GATA6 levels in a cell correlate with the average level expressed in its neighbours, resulting in clusters of cells with similar levels. FGF/MAPK signalling inhibits NANOG expression level clusters, which in turn, likely via NANOG inhibition on GATA6, promote GATA6 clusters. In mid blastocysts, the local patterns are comparable to those in early blastocysts and a global pattern starts to arise in NANOG expressing cells. In late blastocysts, the cells are segregated into two distinct cell groups and show a clear global pattern: NANOG expressing cells are located closest to the ICM centroid, while GATA6 expressing cells are away from the centroid. Hence, expression patterns occur already in early blastocysts, evolve in mid blastocysts and resolve in late blastocysts before the embryo implants. Grey represents NANOG-GATA6 co-expressing cells, purple represents NANOG expression in cells, and green represents GATA6 expression in cells.

In this study, we present a single cell quantification study, which includes three-dimensional neighbourhood analyses to evaluate how NANOG and GATA6 expressing cells are positioned within the ICM with respect to local and global features during cell fate decisions in mouse embryos. The cell neighbourhood is defined by the levels of fate markers expressed by the cell and its neighbours, the number of neighbouring cells and the population type of the cell and its neighbours. We also study a global positional feature by calculating the position of the cell relative to the ICM centroid. These novel three-dimensional analyses allow us to propose a model of how Epi and PrE fates arise from the early blastocyst based on cell neighbourhood descriptors and relative cell position dependant on FGF/MAPK signalling (Fig 9).

### Three-dimensional cell graphs provide local cell neighbourhood

Cell fate decisions rely on groups (communities) of cells showing a coordinated and collective behaviour to achieve the determined fate [43]. Hence, the features of an individual cell have to be put into context of the cell neighbourhood. This need for investigating small groups of cells has also been identified in other developmental contexts [44]. Despite advancements in three-dimensional imaging of developing tissues, investigating the local cell neighbourhood features remains challenging. Even high-end imaging and image analysis protocols focus on the nucleus [45] and rarely include the cell membrane [46], because the number of markers is restricted and the segmentation methods of three-dimensional membrane structures are only slowly evolving [47]. We have shown that the Delaunay Cell Graph (DCG) allows the approximation of the cell neighbourhood from image data for nuclei. Our analysis combines local cell features, providing a good description of the individual cells and the structure of the tissue [12,22,33,36], with correlation analyses, enabling the identification of relationships between two variables [35]. Extending the correlation analysis by the rule-based computer simulation provides a means to describe quantitatively complex three-dimensional population distributions during cell fate decision that is readily applicable to other systems.

To perform a reliable analysis, sufficient amounts of data are required. For the mouse embryos, a robust correlation analysis requires at least 108 cells per measurement. Therefore, a good strategy for future analyses of different signalling pathways might be to first test their features in *in vitro* cultures such as ICM organoids [12] or blastoids [48] and only after a thorough analysis, transfer the results back into mouse embryos.

Here, we obtain the spatial distribution of NANOG and GATA6 expressing cells. To our knowledge, these patterns have not been quantified before. However, we believe they are key to advancing in our understanding of cell differentiation in the preimplantation embryos. There are currently two main models for PrE differentiation [49,50]. The main conceptual difference between them is that the model by Schroter et al. suggests that a bistable system for the NANOG-GATA6 interaction is sufficient, while the second work claims that a tristable system is required. While the model by Schroter et al. focuses on the ratio between Epi and PrE cells [49], the model by Tosenberger et al. has been fitted to the prevailing notion of the expression pattern, i.e. PrE progenitor cells are mainly surrounded by Epi progenitor cells (see Fig 3D in [50]). Our results differ from this proposed pattern. The interesting question is whether a parameter regime exists, for which either of the two models can reproduce the patterns of the three-dimensional local cell neighbourhood.

### Early blastocysts exhibit patterns in local positional features and expression level clustering

Our neighbourhood analyses reveal a pattern in the ICM cells of early blastocysts based on NANOG and GATA6 expression levels. Although most of the ICM cells co-express both markers, the levels of each vary among the different cells. This is reflected in patterns of the local positional features and expression level clustering (Fig 9).

NANOG expression levels in the ICM cells correlate with the cellular arrangement. In early embryos, cells with 8 to 10 neighbouring cells display highest NANOG levels. Several mechanisms could link the positional information to NANOG expression levels. Our results are consistent with previous results of mechanical cues inducing high expression of NANOG in the central cells and their differentiation into Epi cells [51]. Furthermore, it has been shown that the spatial confinement of cells in a three-dimensional microenvironment results in the maintenance of pluripotency even in the absence of LIF [52]. In the early mouse embryo, we might observe a similar effect. The mechanical cues might be sensed via Hippo signalling which has been involved in interpreting positional information (reviewed in [53]). Hippo signalling is clearly determining the first fate choice (TE versus ICM) in the mouse embryos [54,55] and the second fate choice (Epi versus PrE) is linked to the first one [56]. What we observe here might be a reflection of this: first and second cell fate decisions being entwined and Hippo signalling being involved in Epi formation as recently shown [57]. In this study, the authors show attenuation of Hippo signalling promoting nuclear accumulation of YAP in the forming epiblast. In the light of our results, it is plausible that the attenuation of the Hippo signalling might start in those cells having high NANOG levels and around 9 neighbours, and hence contribute to epiblast differentiation.

For GATA6, expression level clustering is observed. The expression levels are independent of the cell localization within the ICM. The functional relevance of the clustering effect might be to ensure an early coordinated PrE cell behaviour during their migration in later stages to occupy their final position at the blastocoele. Recent modelling results for cell population development in ICM organoids show that clonal expansion can play a role in clustering [58]. In addition, the substantial cellular rearrangements taking place during preimplantation development [28,59] might have a positive effect on cell fate clustering. Our results using *Nanog* mutant embryos further indicate a direct or indirect regulation of GATA6 expression level clustering by NANOG. Since PrE fate is regulated through FGF/MAPK signalling (reviewed in [60]), this pathway might also be involved in regulating the spatial distribution of GATA6 and NANOG expressing cells (see below).

The three-dimensional analyses of two independent data sets show a qualitative agreement between the results. The quantitative disagreement between the correlation values obtained are due to the mathematical properties of the correlation coefficient [35,38] and not uncommon in the literature [61]. We have previously used correlations between NANOG and OCT4 levels in individual cells as a pluripotency measurement of mouse embryonic stem cell (mESC) populations. The correlation values decrease as cells differentiate. In this scenario, there are quantitative differences between different wild-type cell lines (Tg2A vs *Tcf3*^*+/+*^) cultured under the same conditions, which coincided with them having different pluripotency potential. Given the embryonic origin of the mESCs, one can envisage a similar situation in the mouse early embryos. The quantitative differences found in the correlation values between the data sets might be due to differences in the variability of the measurements related to the experimental setups or embryos being in slightly different developmental stage. All data sets were staged according to total cell number, the usual method to stage preimplantation embryos. However, this method might not be a reliable timing mechanism and variations can have a quantitative effect on accuracy similar to what we observe here [62]. Indeed alternative ways of measuring developmental timing have been proposed, like the number of DP cells we proposed in ICM organoids, (continuous staging; [12]), or morphogenetic events in rabbit embryos [63].

The theoretical model allows us to break down the complex three-dimensional population pattern into two simple rules with three parameters. Eliminating one of the rules reproduces the *Nanog* mutant situation and the experimental results agree with the simulation. Hence, the population composition in ICMs of early embryos can be derived from the local neighbourhood features.

Altogether, our results are consistent with positional information impinging on cell fate decision in early blastocysts. This, together with previous results, suggests that very early in development, when ICM cells are co-expressing NANOG and GATA6, the two transcription factors as well as FGF/MAPK signalling impact on their expression levels and that the cells are already deciding about their fate.

### A global pattern of NANOG and GATA6 expression in the ICM starts arising in mid blastocysts

Our results show that the global positional features (see Terminology Box) of NANOG and GATA6 in early blastocysts do not show a pattern. This lack of a pattern might allow for the previously observed plasticity during the cell fate decision process [22,28,64,65].

Starting in mid and fully established in late blastocysts, once the decision has been made and fate reversal does no longer occur, we see the expected distribution. Higher NANOG level expressing cells are located at the centre of the ICM and higher GATA6 level expressing cells are at the edge. Hence, our results indicate that the cell fate specification does not correlate with the global position of a cell in the ICM. Only once the cell fate is specified, the cells arrange in a global pattern.

Our results show that *Nanog* is involved in the evolvement of the global pattern in late blastocysts as in its absence the GATA6 pattern disappears. A previous study has shown that differential adhesion between ICM cells and directional movement, together with differential adhesion between ICM and TE cells or forces pushing from the blastocoele are responsible for the final distribution of Epi and PrE cells in late blastocysts [59,66]. According to the Krupinski study, Epi cells would have stronger adhesion between them than with other cell types, while PrE cells would show a directed movement towards the blastocoele. In this context, the absence of *Nanog* would be interpreted as absence of differential adhesion. This scenario would result in a lack of global pattern, which is in agreement with our data.

### FGF/MAPK signalling affects NANOG and GATA6 expression level clustering

It has been shown that FGF/MAPK signalling is the main signalling pathway involved in Epi versus PrE differentiation (reviewed in [60]). Our three-dimensional analyses of FGF/MAPK signalling inhibitor treated embryos also implicates this pathway in the regulation of the three-dimensional local clustering of NANOG and GATA6 expressing cells. We did not obtain a clear effect of this pathway on the local or global positional features. There are several explanations for this: FGF/ERK signalling is not involved in establishing the global pattern, the long-term culture of embryos affects their global pattern, or the embryos analysed here are in a different stage from the freshly flushed ones (more than 150 cells *versus* less, respectively). We favour the explanation of an issue with the long-term culture of embryos since cultured embryos until E4.5 stage clearly do not have the same shape (spherical) as freshly flushed E4.5 embryos (prolate; compare embryos shown in Fig 1B stages 120–150 and >150 to those shown in Fig 2C in [22]). Furthermore, it has been shown that embryo culture delays their development [67,68]. The change in the overall shape of the cultured embryos together with their delay are key differences that should not be overlooked as they will affect the global pattern and we believe both differences are at the core of the results obtained.

Our results, together with previous work, allow us to suggest the following series of events during cell fate decision making in early embryos. *Fgf4* expression is directly regulated by the OCT4-SOX2 dimer (as is *Nanog*, [69,70]), and it is expressed in a subpopulation of ICM cells [8,9,11]. We hypothesize that the secretion of FGF4 starts, or is higher, in the subpopulation of NANOG positive cells with 8 to 10 neighbours. Binding to (mainly) FGFR1 presented in the cells activates the signalling pathway [9,10]. Activation of the pathway has opposite effects: it promotes autocrine NANOG degradation via ERK1 phosphorylation and paracrine GATA6 upregulation via ERK1/2 phosphorylation [10,23,71,72]. Autocrine NANOG inhibition might result in the low correlation found in NANOG levels between neighbouring cells. Upregulation of GATA6 will result in the upregulation of FGFR2 expression in those cells as suggested by ChIP-seq experiments [73]. GATA6 expression level clusters might be due to neighbouring cells receiving similar levels of FGF4, hence activating the downstream effectors to a similar extend. The clusters might also be related to directed active movement of the cells at this stage towards the cavity [3]. The effect of FGF/MAPK signalling on the GATA6 clustering could be related to both, as signal inhibition results in decreased correlations and reduced cell movement of ICM cells [28].

As *Fgf4* expression depends on NANOG [4], the decrease in GATA6 expression level clustering observed in the absence of NANOG reinforces the idea that the three-dimensional cell neighbourhood features are regulated by FGF/MAPK signalling. However, this poses the question of how FGF4 is propagated extracellularly once secreted and how its activity is inhibited in the direct neighbours. One possibility is that it is via differential expression of heparan sulfate (HS) chains, which has been associated with heterogeneous di-phosphorylated Erk at this embryonic stage [74]. Another alternative is changes in the internalization and spreading related to endocytosis rates as shown for FGF8 in zebrafish embryos [75].

In summary, we propose that the coordinating mechanism behind the three-dimensional distribution of NANOG and GATA6 expressing cells in early blastocysts is FGF/MAPK signalling. However, we cannot rule out that other major signalling pathways involved in patterning fields or groups of cells, such as Notch, Wnt, BMP, Hippo or EGF might also have an input [76]. In support of this, there are reports of Notch signalling involved in early mouse development [29,77,78], as well as Wnt signalling [79,80], BMP signalling [81,82], p38/MAPK signalling [83] and EGF signalling [84]. In the light of our results, it will be important to revisit how these signalling pathways might be involved in cell fate decisions in early blastocysts, investigating how they affect the local cell neighbourhood features and the global positional feature within the ICM.

## Supporting information

S1 Text Full description of the data analysis. (PDF)

S1 Fig Schematic diagram of the image analysis and data pre-processing (data I). **Step 1:** The confocal images of the fixed embryos are segmented with MINS to obtain the centroid, the cell type (ICM or TE) and the mean NANOG and GATA6 expression levels of a nucleus. Subsequently, the cell fate assignment to TE or ICM is manually checked. **Step 2:** Data I, provided in four different independently imaged batches, are aligned according to their thresholds for high NANOG and high GATA6 expression levels. Top: Scatter plots showing the raw values for NANOG (horizontal axis) and GATA6 (vertical axis) levels in ICM cells in early, mid and late blastocysts (left, centre and right, respectively) in arbitrary units (a.u.). Each dot represents the levels in a single cell from 26 early, 4 mid and 15 late blastocysts. Further details on the number of embryos and cells analysed are in S1 and S2 Tables. Bottom: Scatter plots showing NANOG (horizontal axis) and GATA6 (vertical axis) levels in ICM cells in early, mid and late blastocysts (left, centre and right, respectively) after aligning the data sets. Dashed lines represent the threshold levels for NANOG and GATA6. **Step 3: (i)** Illustration of the cell position rescaling for one embryo to account for slight squeezing along the z-axis due to the mounting. **(ii)** Illustration of the Delaunay Cell Graph (DCG) for this embryo. Lines represent neighbourhood relationship between cells. **Step 4:** Selecting the cells that are relevant for the analyses. We analyse the features of the ICM cells and as neighbours we include the ICM cells and the TE cells that are neighbouring at least one ICM cell. Illustration of the selected cells and the DCG (left), and of the table containing the relevant data (right). See S1 Sup. Info. text for further details.
(PDF)

S2 Fig Population analyses (data I). **(A-C)** Population analysis of individual embryos staged by total cell number (early: 32–64 cells, mid: 65–90 cells, late >90) of all ICM cells (A), TE cells with ICM neighbouring cells (included in subsequent analyses, B) and all TE cells (C). Error bars indicate the standard errors of the means. Details on the number of embryos and cells analysed are in S1 and S2 Tables.
(PDF)

S3 Fig Local and global positional features of ICM cells according to their population type (data I). **(A)** Mean number of neighbouring cells (vertical axis) versus the distance to the ICM centroid (horizontal axis) of the indicated cell populations in ICMs of early (grey), mid (yellow) and late (blue) blastocysts. Shaded regions indicate the standard errors of the means. **(B)** Scatter dot plot showing the total number of neighbouring cells of DN, DP, Epi progenitor and PrE progenitor cells in ICMs of early (left panel), mid (centre) and late (right) embryos. Mann-Whitney test with Bonferroni correction gives no statistically significant results in all the comparisons (p<0.05). The red horizontal line indicates the mean values. Details on the number of embryos and cells analysed are in S1 and S2 Tables.
(PDF)

S4 Fig Neighbour composition statistical analyses (data I). Tables showing the statistical test results (z-test) for a pairwise comparison of cell neighbour type for each cell population type in the different developmental stages for data set I. *: p<0.05 (with Bonferroni correction); ns: not significant. E.g. a DN cell has significantly more TE neighbours than DN neighbours. Details on the number of embryos and cells analysed are in S1 and S2 Tables.
(PDF)

S5 Fig Extended correlation analysis (data I). **(A-B)** Spearman’s correlation coefficients for GATA6 levels of a cell and the median NANOG levels of its neighbours **(A)** and NANOG levels of a cell and the median GATA6 levels of its neighbours **(B)** at different embryonic developmental stages. **: p<0.01 Mann-Whitney test with Bonferroni correction for comparison with the null model (see S1 Text for further details). The error bars represent the standard errors calculated by bootstrap sampling the experimental data 100 times. Striped boxes indicate populations composed by less than 108 cells. In those cases, no statistical analysis was performed. **(C-F)** Scatter dot plots of the expression levels of the indicated fate markers in individual cells (horizontal axis) and the indicated median fate marker levels of their neighbours (vertical axis) in the specified cell population types and developmental stages in arbitrary units (a.u.). Each dot represents a cell. Only those populations composed of more than 108 cells are shown. The Spearman’s correlation coefficients are shown (r). Details on the number of embryos and cells analysed are in S1 and S2 Tables.
(PDF)

S6 Fig Visualisation of relation of number of neighbours of a cell to its NANOG levels in data I. Three-dimensional Illustrations for number of neighbours and NANOG level for all ICM cells in all early blastocysts of data I. For each embryo two illustrations are shown: the normalised absolute difference of the number of neighbours of a cell to nine (left) and the normalised expression level of NANOG (right). Both values are normalised to the maximum in each embryo. I.e. Cells with nine neighbours and maximum NANOG level are shown in red in both images.
(PDF)

S7 Fig Extended positional features (data I). **(A)** Mean level of NANOG (left) or GATA6 (right) (vertical axis) versus the number of neighbours (horizontal axis) for the null model simulation of ICM cells of data I in early (grey), mid (yellow) and late (blue) blastocysts. Error bars indicate the standard errors of the means. **(B)** Mean level of NANOG (left) or GATA6 (right) (vertical axis) versus the distance to the ICM centroid (horizontal axis, binned in 5 μm groups) for the null model simulation of ICM cells of data I in early (grey), mid (yellow) and late (blue) blastocysts. Shaded regions indicate the standard errors of the means. **(C)** Tables summarizing the statistically significant results of the Mann-Whitney statistical tests with Bonferroni correction comparing NANOG or GATA6 levels at the indicated positions relative to the ICM centroid; *: p<0.05. Related to Fig 4C and 4D.
(PDF)

S8 Fig Local neighbourhood features for the [22] data set (data II). **(A, B)** Mean level of NANOG (A) or GATA6 (B) (vertical axis) versus the number of neighbours (horizontal axis) for ICM cells in early (grey), mid (yellow) and late (blue) blastocysts. The tail of the graph for early embryos in (A) is due to DP cells with high NANGOG levels and a large number of neighbours. The error bars indicate the standard errors of the means. **(C-F)** Scatter dot plots of the expression levels of the indicated fate markers in individual cells (horizontal axis) and the indicated median fate marker levels of their neighbours (vertical axis) in the specified cell population types and developmental stages in arbitrary units (a.u.). Each dot represents a cell. The colours represent different embryos. Only those populations with at least a moderate correlation strength, i.e. correlation coefficient greater than 0.4, are shown. **(G, H)** Mean level of NANOG (G) or GATA6 (H) (vertical axis) versus the distance to the ICM centroid (horizontal axis) for ICM cells in early, mid and late blastocysts. Mann-Whitney test between the indicated levels; **: p<0.05. For simplicity, only selected significant results are indicated for NANOG levels in mid and late embryos, GATA6 levels in late embryos, full statistical results are shown in S9 Fig. The shaded regions indicate the standard errors of the means. Details on the number of embryos and cells analysed are in S1 and S2 Tables.
(PDF)

S9 Fig Statistical analysis of marker expression levels versus distance to the ICM centroid for the [22] data set (data II). Tables summarizing the results of the Mann-Whitney statistical tests with Bonferroni correction comparing NANOG (A-B) and GATA6 (C) levels at the indicated positions relative to the ICM centroid in mid (A) and/or late blastocysts (B-C); *: p<0.05, ns: not significant. Related to S8G and S8H Fig. Details on the number of embryos and cells analysed are in S1 and S2 Tables.
(PDF)

S10 Fig Extended results of *Nanog* mutant analysis (data III and IV). **(A)** Scatter dot plot showing GATA6 expression levels in the indicated cell populations and developmental stages in *Nanog*^*+/+*^ or *Nanog*^*+/-*^ and *Nanog*^*-/-*^; **: p<0.01 Mann-Whitney test with Bonferroni correction. The red horizontal line indicates the mean values. **(B)** Table summarizing the results of the Mann-Whitney statistical tests with Bonferroni correction comparing GATA6 levels at the indicated positions relative to the ICM centroid in *Nanog*^*+/+*^ or *Nanog*^*+/-*^ embryos at the indicated positions; *: p<0.05, ns: not significant. Details on the number of embryos and cells analysed are in S1 and S2 Tables.
(PDF)

S11 Fig NANOG and GATA6 expression levels upon PD03 treatment for 24 h or 20 h from [22] (data V-VIII). **(A-B)** Scatter dot plots showing the expression levels of NANOG in Epi progenitor cells of embryos cultured for 24 h (A) or 20 h (B) with control (grey) or PD03-containing (red) media; **: p<0.01 Mann-Whitney test with Bonferroni correction. **(C-D)** Scatter dot plots showing the expression levels of GATA6 in PrE progenitor cells of embryos treated for 24 h (C) or 20 h (D) with PD03; **: p<0.01 Mann-Whitney test with Bonferroni correction; ns: not significant. In all plots, the red horizontal line indicates the mean values. Details on the number of embryos and cells analysed are in S1 and S2 Tables.
(PDF)

S12 Fig Local and global positional features upon PD03 treatment for 24 h or 20 h from [22] (data V-VIII). **(A-B)** Mean level of NANOG (vertical axis) versus the number of neighbours (horizontal axis) for ICM cells in embryos cultured for 24 h (A) or 20 h (B) with control (grey) or PD03-containing (red) media. **(C-D)** Mean level of GATA6 (vertical axis) versus the distance to the centre of the ICM (horizontal axis) for ICM cells in embryos treated for 24 h (C) or 20 h (D) with PD03. **(E-F)** Mean level of NANOG (vertical axis) versus the distance to the centre of the ICM (horizontal axis) for ICM cells in embryos treated for 24 h (E) or 20 h (F) with PD03. In all plots, error bars or shaded regions indicate the standard errors of the means, respectively. Details on the number of embryos and cells analysed are in S1 and S2 Tables.
(PDF)

S1 Video z-stack of early (1), mid (2) and late (3) embryos comparing DGC neighbour assignment and fluorescent immunostaining. The left panels show membrane and/or nuclear staining. The yellow dots indicate DCG calculated neighbouring cells of the cell with an encircled number, that number indicates its number of neighbours; numbers in other cells indicate the number of neighbouring cells of that cell. The right panels show the original confocal images of the embryos shown, stained for NANOG (magenta), GATA6 (green), DAPI (blue) and β-catenin (membrane, red).
(7Z) Click here for additional data file.

S1 Dataset (7Z)

S1 Table Numbers of analysed embryos. (PDF)

S2 Table Numbers of analysed cells. (PDF)
