# Supplementary material for: The transition from local to global patterns governs the differentiation of mouse blastocysts
Source: PLoS One. 2020 May 15;15(5):e0233030. doi: 10.1371/journal.pone.0233030 (PMC7228118; doi:10.1371/journal.pone.0233030)
Supplement: S1 Text — (PDF) [file pone.0233030.s001.pdf]

S1 Supplementary Information text: Full description of the data analysis

In this supplementary information, we provide a full description of the different steps of our data analysis. This includes the arguments for the choice of a particular approach as well as the sensitivity of the output of each approach to its input parameters. Investigating the sensitivity of a method increases our understanding of the relationship between input and output variables. Furthermore, it provides information on the robustness of the approach.

In the following, we consider the four main parts of our analyses:

1. Normalization of expression values and classification of cell populations
2. Determining cell neighbours with the Delaunay Cell Graph (DCG)
3. Correlations of expression levels of neighbouring cells
4. Rule-based simulations of population composition in ICM of early blastocysts

1. Normalization of expression values and classification of cell populations

The imaging data for the embryos was generated in four batches corresponding to different imaging sessions and/or stainings. The mounting of the embryos for imaging resulted in a slight squeezing along the z-axis of the image and hence extension along x and y (Fig S1, Step 3(i)). We checked for fluorescence intensity decay along the z-axis for each batch. As this decay was minimal due to the mounting of the embryos, intensity adjustment along z was not performed (Fig 1).

We assumed that the embryos that do not have fully segregated epiblast and primitive endoderm should be spherical (early and mid blastocysts). Based on this assumption, we calculated the deviation from sphericity for each of these embryos and rescaled the coordinates of the cell nuclei to obtain spherical embryos. Embryos with segregated epiblast and primitive endoderm have hatched from the zona and are elongated (late blastocysts). To rescale the coordinates of these late stage

embryos, we calculated for each batch the median rescaling factors for x, y and z of the early and mid blastocysts and applied these to the late blastocysts.

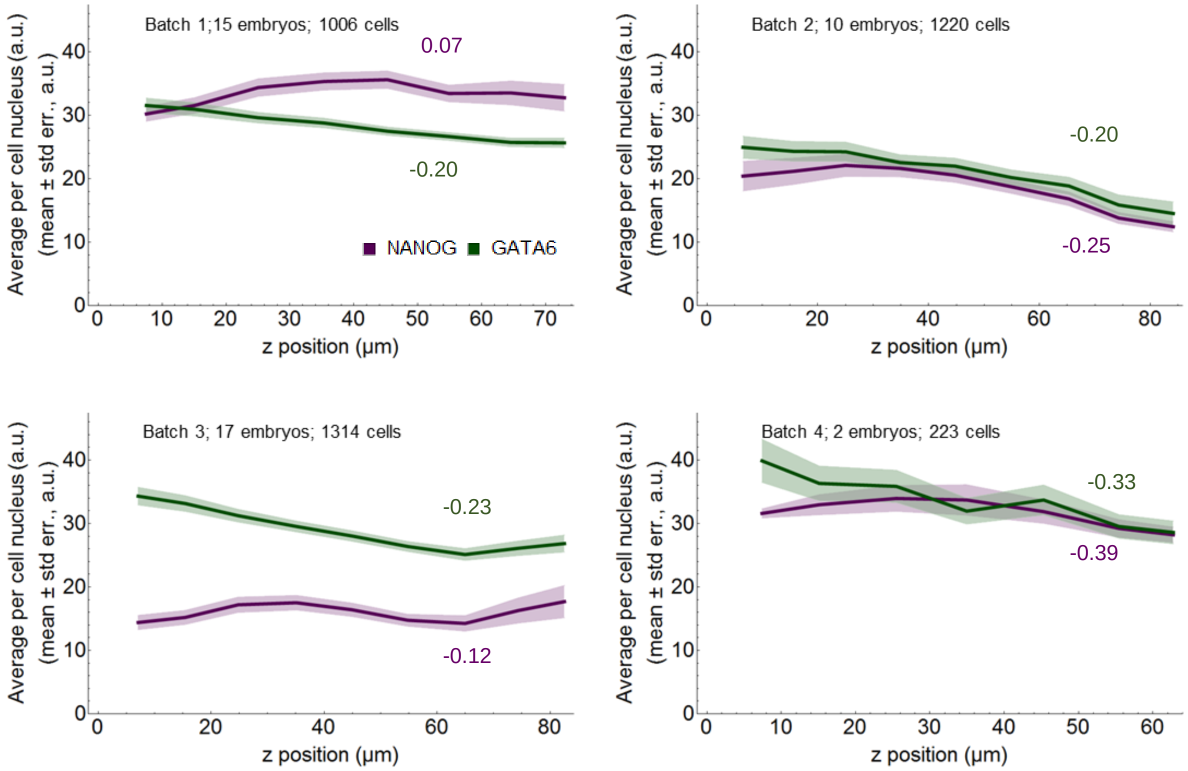

Fig 1: Fluorescence intensity distribution along the z-axis. Mean level of NANOG (purple) or GATA6 (green) (y-axis) fluorescence intensity in each cell (ICM and TE) versus its z position within the imaged z-stack in each of the four imaged embryo batches. The z position data are binned in 10 μm intervals. The shaded regions display the standard error of the mean. Batch, embryo and total cell numbers are indicated. Coloured numbers indicate the Spearman correlation values between NANOG (purple) and GATA6 (green) levels. Note that all values show a very weak or weak correlation indicating no evident decay of fluorescence intensity in deeper z positions.

Plotting the mean GATA6 expression levels versus the mean NANOG expression levels for all nuclei in the four imaging batches, we observed a shift in the data related to the batch number (Fig S1, Step 2). To align the data obtained from the four independent sessions, we established thresholds for NANOG and GATA6 expression for each batch, based on the data distribution in late stage embryos, where Epiblast (Epi) and Primitive Endoderm (PrE) are completely separated and no double positive (DP) cells occur. The thresholds were manually adjusted. The criterion was to determine the minimal

NANOG and GATA6 value, respectively, such that there are no double positive cells in late blastocysts (see Fig 3).

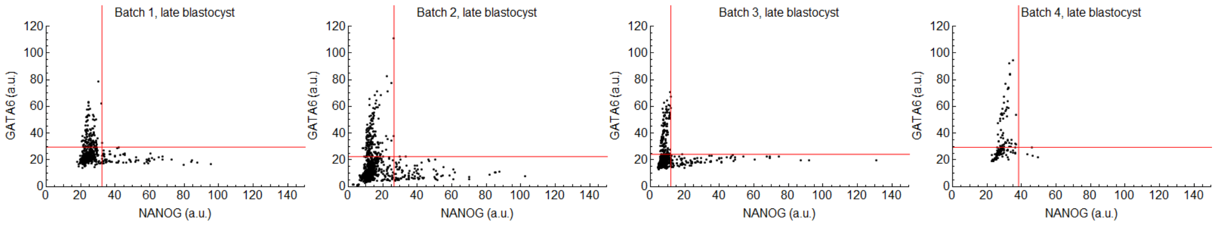

Fig 3: Population thresholds. Raw data for NANOG and GATA6 expression in single cells in arbitrary units (a.u.) in late embryos for the four batches (black) and the manually set thresholds to determine the four populations (red).

Based on the thresholds, we linearly shifted the data of all batches and all stages, such that all thresholds fall on top of each other. Since the range of the data does not vary much between batches, we consider such a linear transformation most appropriate. This changes the absolute intensity levels for each embryo but it does not change the relative intensity values in an embryo, which is the value that is relevant for our analysis. Based on the thresholds for GATA6 and NANOG, all cells were classified as double negative (DN: N- and G-), NANOG+/GATA6- (N+G-), NANOG-/GATA6+ (N-G+) and double positive (DP: N+, G6+). We applied the same method to the *Nanog* mutant data set to align the thresholds obtained from the data obtained in the five imaging sessions.

We also tested the k-means clustering used in [1] to determine the thresholds for our WT embryo data set as well as for the *Nanog* mutant data sets. Unfortunately, for the mutant data set, the clustering approach gave unreasonable results, including a large proportion of DN cells. Therefore, we decided to use the manually adjusted approach that works for both cases.

For the [1] data set, the imaging was performed in small dishes that didn´t require the mounting of the embryos, hence a rescaling was not required. Instead, the data set was corrected for the decrease in intensity along z. Furthermore, Saiz et al. employed a k-means clustering for the population assignment of their data. We took the corrected data set and population assignment from

[1] to perform our neighbourhood analyses. We noticed that there were some oversaturated nuclei images and hence excluded all NANOG and GATA6 levels from the distribution that were two standard deviations away from the respective mean. The remaining analysis was analogous to our data.

2. Determining cell neighbours with the Delaunay Cell Graph (DCG)

We recently proposed two approaches to model the cell neighbourhood [2]: the Proximity Cell Graph (PCG), which provides a purely distance-based description of the cell neighbourhood and the Delaunay Cell Graph (DCG), in which neighbourhood is determined by the Delaunay triangulation. The Delaunay triangulation and its dual, the Voronoi tessellation, are routinely used to approximate the nearest neighbours of a cell [2–4]. The Delaunay cell graph (DCG) is given by $DCG(V, E)$ where V is the vertex set and E is the edge set of the graph. An edge $(u, w) \in E$ exists between two vertices $u \in V$ and $w \in V$ if the corresponding points are connected by a line in the Delaunay triangulation and the Euclidean distance between u and w is less than a given threshold, which we chose as 30 µm (three times the average diameter of a cell nucleus). To validate that the distance between the centroids of two neighbouring cells is at least 10 µm, we calculated this distance between all neighbouring cells in the ICM (and their TE neighbours) for all embryos in data set I and II (Fig 3). These values fall within the reported segmentation errors obtained with MINS [5], increasing in later embryos.

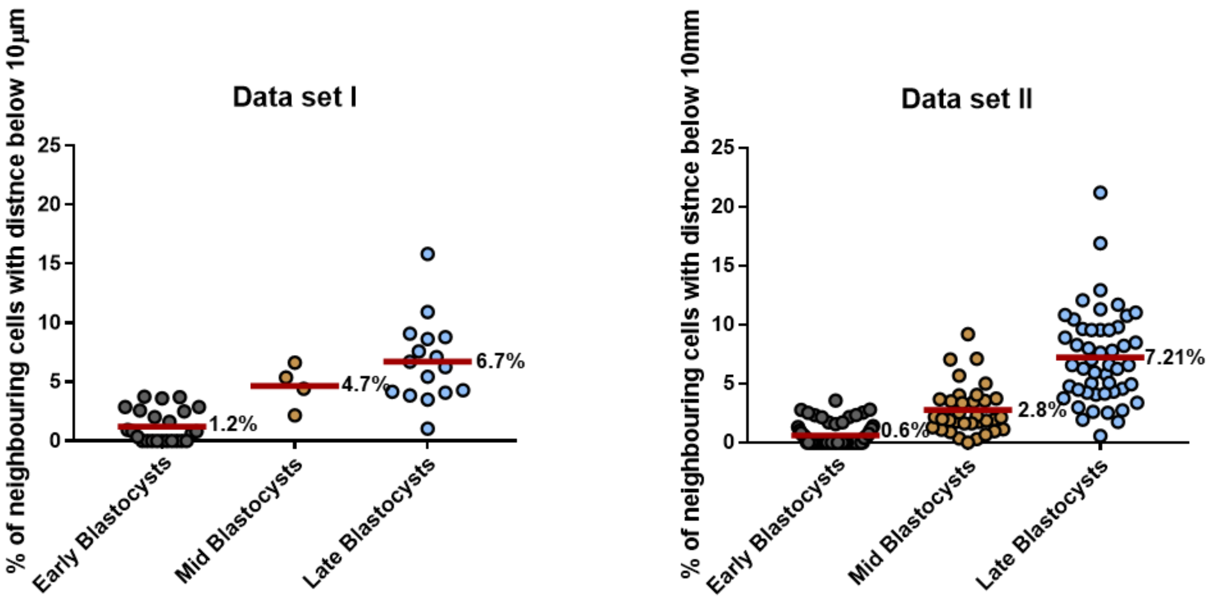

Fig 3: Percentage of cells with a distance below 10 μm in data set I and II. Each dot represents the percentage of distances of a cell to all its neighbours that fall below 10 μm in one embryo. The horizontal red line represents the average percentage within each developmental stage (numerical value is also shown).

In a preliminary study to decide whether to use the Proximity Cell Graph (PCG) and/or the Delaunay Cell Graph (DCG) in mouse embryos, we generated a number of artificial shapes (ball, oblate spheroid, prolate spheroid, cuboid and box) and analysed the number of edges versus the number of vertices. As expected, we found that the number of edges in the PCG increases unreasonably with increasing number of vertices [2]. Furthermore, by definition, the PCG is completely dependent on the cut off length. PCG approximation was used in a recent study on a model for early cell lineage specification in mouse embryos with neighbour type simulations [6]. The authors assume in their simulations that the cells are non-overlapping spheres with a certain radius and a cut off length 1.2 times the sum of the radii of the two cells. This is essentially if the spheres touch or almost touch. For a tissue with polygonal cells of different sizes like the ones found in mouse embryos, it is not trivial to determine an appropriate cut off length to obtain the nearest neighbours. The DCG is less sensitive to the cut off length and for cells in the centre of the embryo like in the ICM, the cut off length is irrelevant unless it is unreasonably small. Therefore, we decided to analyse the embryo data using the DCG.

We employed the DCG on the pre-processed nuclei centre of mass. For a given cell (vertex in the cell graph), the neighbouring cells are represented by the vertices that are connected through edges. In all our analyses, the set of neighbouring cells consists of all ICM cells and the TE cells that are neighbours to at least one ICM cell (Fig S1, Step4).

To evaluate the neighbour assignment of the DCG in the mouse embryos, we compared the results for the ICM cells of four early, three mid and two late embryos to a manual assignment included in our data set (Fig 4, and see Sup. Videos 1-3). For the manual assignment, we considered cells as neighbours if they are touching, identified by the membrane staining. The manual assignment only provides an indication. Judging the three-dimensional neighbourhood of the cells from slices of two-dimensional images is very difficult and gets even more challenging as the cell density and the irregularity of the cellular geometry increase with stage. We find that the accuracy of DCG compared to the manual assignment is 91 % (early), 82% (mid) and 83% (late) (see Table 1). We also found that 0 (early), 3 (mid) and 13 (late) cells were not detected as neighbours by the automatic assignment compared to the manual assignment due to errors in the segmentation method used (MINS). Hence, if the cells were segmented correctly, the DCG did not miss a neighbourhood relationship. We conclude that for all stages, the DCG provides a robust description of the local cell neighbourhood in the ICM and a satisfactory approximation of which cells are touching.

Table 1: Comparison of DCG neighbours to manual assignment of touching cells

	Early (n=4)	Mid (n=3)	Late (n=2)
ICM cells	50	67	79
DCG neighbours	543	751	1031
Manual neighbours (touching)	495	617	851
Accuracy: Manual/DCG neighbours	0.91	0.82	0.83

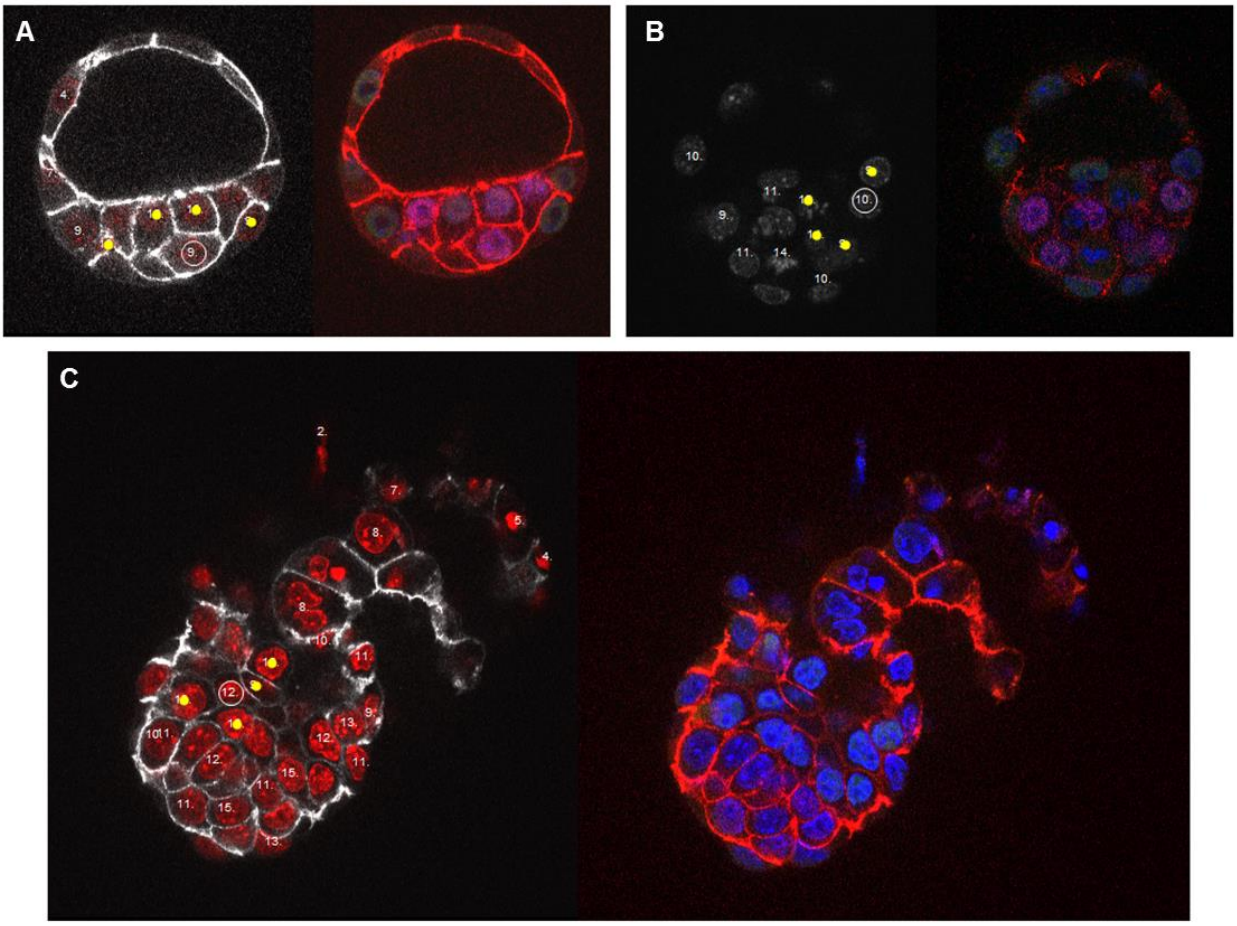

Fig 4: z Sections of early (A), mid (B) and late (C) embryos comparing DGC neighbour assignment and fluorescent immunostaining. The left panels show membrane and/or nuclear staining. The yellow dots indicate DCG calculated neighbouring cells of the cell with an encircled number, that number indicates its number of neighbours; numbers in other cells indicate the number of neighbouring cells of that cell. The right panels show the original confocal images of the embryos shown, stained for NANOG (magenta), GATA6 (green), DAPI (blue) and β-catenin (membrane, red). Note that the embryos are upside down and not all the neighbours of the indicated cell are located in the same z section. See Sup. Videos 1-3 for the complete z-stack.

<u>Sensitivity of the DCG</u>

We investigated the sensitivity of the number of neighbours provided by the DCG with respect to cell density. We considered a ball of radius 50 μm and randomly filled it with non-overlapping spheres of radius 5μm to represent the cells. For these simulated cells, we generated the DCG. The procedure was repeated ten times for cell numbers ranging from 10 to 400 in steps of 10, resulting in

cell densities ranging from 1.9×10^{-5} cells/µm³ to 76.43×10^{-5} cells/µm³. For comparison, manual inspection of the ICM cells resulted in a cell density of 4×10^{-5} cells/µm³ in early embryos and 20×10^{-5} cells/µm³ in mid embryos.

For the simulated DCGs, we find that for increasing cell density the average number of neighbours increases and plateaus at around 13 neighbours (Fig 5).

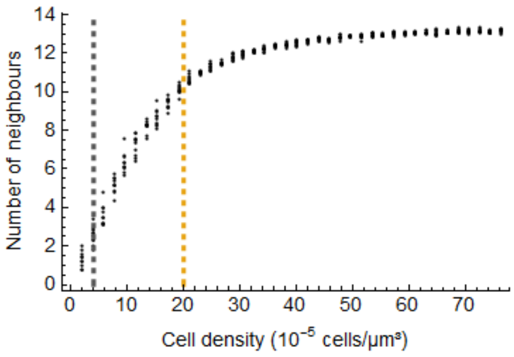

Fig 5: The mean number of neighbours derived from the DCG plateaus for high cell densities. Each dot represents the mean number of neighbours of one simulated DCG. The vertical lines indicate the manually obtained cell densities in ICM of early (grey) and mid (yellow) blastocysts.

3. Correlations of expression levels of neighbouring cells

To relate the expression levels of a given cell to the expression levels of all its neighbours (both TE and ICM), we calculated Spearman's correlation coefficient of the expression levels of a cell and the median expression levels of its neighbours. We chose to use median level in the neighbours in combination with Spearman's correlation coefficient as we reasoned that this measurement was the variable, which made the least assumptions about the type of signals that might be regulating the observed correlations. Furthermore, the median provides a more robust measure than the sum of all signals as the median goes up slower and is also less sensitive to outliers than the sum. Spearman's correlation coefficient does not require normally distributed data.

To determine whether the obtained correlations are statistically significant, we performed a bootstrap resampling of the correlation coefficients of our data and compared the result with

correlation coefficients of a null model. For the bootstrapping, we resampled the experimental data to create 100 different data sets.

Sensitivity of the correlation analysis:

Spearman's rank correlation coefficient is the Pearson correlation coefficient after the two variables have been separately transformed to ranks while retaining their pairing [7]. The value of the correlation coefficient is affected by a number of factors, in particular the sample size [8]. Therefore, we explored the sensitivity of the correlation value to the number of cells analysed. We know that for a random distribution, the correlation value is zero. To test whether this analysis might be affected by the topological properties of small DCG given the specific constraints on cell number, we used 100 artificially generated DCGs. These artificial DCGs consisted of cell numbers 10 to 400 in steps of 10 and randomly assigned expression levels to each cell based on the uniform distribution over [0,1]. For each DCG, we then calculated the correlation value and determined the mean correlation value for DCGs with the same cell number. We find that if the number of cells analysed increases, the mean correlation coefficient approaches zero (Fig 6). Hence, if the number of cells is large enough, the correlation analysis is consistent.

Next, we investigated how the number of cells in the analysis is linked to the deviation of the mean correlation from zero. Fitting the function $f(x) = -\frac{a}{x} - b$ resulted in a=2.19 and b=0.0098. Based on $f(x)$, we estimated that on average, we need at least 108 cells in the analysis to obtain at most 3% deviation (Fig 6). Less cells will lead to more noise in the correlation analysis, while more cells will increase the precision. We chose 3 % as the threshold, since this is the point where the functions levels off.

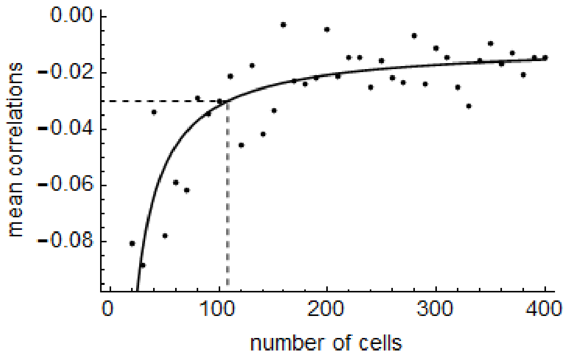

Fig 6: More than 108 analysed cells results in an average deviation of less than 3%. Mean correlation coefficients for 100 artificially generated DCGs of 10 to 400 cells in steps of 10 with expression levels drawn from the uniform distribution over [0,1] (dots). The continuous line indicates the fitted curve $f(x) = -\frac{2.19}{x} - 0.0098$ and the intersection of $f(x)$.The 3% threshold is marked by the dashed line.

The ICM has 20 ± 1 cells in early, 24 ± 1 cells in mid, and 45 ± 4 cells in late embryos. Due to these small numbers, analysing the correlations individually in each embryo does not provide reliable results. Hence, we pooled the data for all cells, expecting a reliable result if the number of pooled cells is at least 108.

<u>Null model for correlations:</u>

The correlation analysis for the experimental data results in non-zero values. To test whether these results might be affected by specific constraints on NANOG/GATA6 distributions, we investigated whether the correlation values are significantly different from those of a null model. For the null model, we assumed the embryo geometry is given by the experimental data, hence we used the measured coordinates of the cells. The expression levels of the TE cells were also based on the experimental data. To assess the effect of NANOG/GATA6 distribution, we generated several different models using different assignment rules for the NANOG and GATA6 values of the ICM cells:

- Random model 1: The NANOG and GATA6 values of the ICM cells are randomly drawn from the uniform distribution over [0,1].
- Random model 2: The values of the ICM of each embryo are shuffled randomly.

• Random model 3: We generate the distributions for NANOG and GATA6 from all ICM cells of all embryos at all stages. The values of an ICM cell are randomly drawn from these distributions.

• Random model 4: We generate the distributions for NANOG and GATA6 from all ICM cells of all embryos of a given stage. This results in six distributions one for each stage for NANOG and GATA6, respectively. For a cell in the ICM of a given embryo, the values for NANOG and GATA6 are randomly drawn from the corresponding distribution depending on the stage of the embryo.

• Random model 5: The values of the ICM cells of all embryos in each stage are randomly shuffled.

We generated each model for all the embryos in our data set, pooled all the cells in the ICM from one stage and calculated the correlations of these cells with their neighbours both for NANOG (Table 2) and GATA6 (Table 3). This procedure was repeated 100 times. We expect very low correlation values for the random models.

Table 2: Correlation values for different random assignments of NANOG expression (mean ± standard deviation)

**Model**	**Early**	**Mid**	**Late**
Random model 1	-0.0006 ± 0.04	0.15 ± 0.01	0.25 ± 0.003
Random model 2	0.2 ± 0.04	0.04 ± 0.08	0.3 ± 0.05
Random model 3	-0.005 ± 0.05	-0.02 ± 0.1	-0.004 ± 0.04
Random model 4	-0.009 ± 0.04	-0.02 ± 0.1	-0.02 ± 0.04
Random model 5	0.003 ± 0.06	0.002 ± 0.1	-0.01 ± 0.04

Table 3: Correlation values for different random assignments of GATA6 expression (mean ± standard deviation)

Model	Early	Mid	Late
Random model 1<br><br>(identical to above)	-0.0006 ± 0.04	0.14 ± 0.01	0.25 ± 0.003
Random model 2	0.6 ± 0.01	0.4 ± 0.07	0.4 ± 0.04
Random model 3	-0.005 ± 0.05	0.0003 ± 0.1	-0.0001 ± 0.04
Random model 4	-0.001 ± 0.05	-0.02 ± 0.1	0.002 ± 0.04
Random model 5	-0.004 ± 0.05	0.004 ± 0.1	0.006 ± 0.04

We find that Random model 2 exhibits larger correlation values than the other models. This indicates that reshuffling the values of the ICM cell in each embryo individually does not introduce a sufficient randomization, due to the small number of cells in the ICM.

All the other models show similar results with values close to zero as expected from a random model. For all subsequent analyses shown in the main text, we used the method of Random model 3 to calculate the null models for the neighbour correlations, rather than models 1, 4 or 5. The main reasons for this are that Random model 3 relies on the original data (unlike Random model 1) and its calculation is more straightforward, because we only need to consider two distributions for the data (one for NANOG and one for GATA6) rather than six as for Random models 4 and 5.

The correlation values for Random model 3 for the different experimental conditions are summarised in Tables 4 and 5.

Table 4: Correlation values for Random model 3 for wild-type and NANOG mutant analyses (mean ± standard deviation)

	Correlations		
**Experimental condition**	**Early**	**Mid**	**Late**
Our data (NANOG cell, NANOG neighbours)	-0.005 ± 0.05	-0.02 ± 0.1	0.004 ± 0.04
Our data (GATA6 cell, GATA6 neighbours)	-0.005 ± 0.05	0.0003 ± 0.1	-0.0001 ± 0.04
Our data (NANOG cell, GATA6 neighbours)	-0.003 ± 0.04	-0.005 ± 0.1	0.006 ± 0.04
Our data (GATA6 cell, NANOG neighbours)	-0.002 ± 0.05	-0.02 ± 0.1	-0.0004 ± 0.04
Saiz data (NANOG cell, NANOG neighbours)	-0.0005 ± 0.03	0.001 ±0.04	-0.0008 ± 0.03
Saiz data (GATA6 cell, GATA6 neighbours)	-0.002 ± 0.03	-0.008 ± 0.03	0.0001 ± 0.03
Saiz data (NANOG cell, GATA6 neighbours)	-0.004 ± 0.03	-0.002 ± 0.04	-0.0006 ± 0.03
Saiz data (GATA6 cell, NANOG neighbours)	-0.004 ± 0.03	-0.0009 ± 0.03	-0.003 ± 0.03
NANOG mutant analysis, WT and heterozygotes (GATA6 cell, GATA6 neighbours)	-0.0008 ± 0.04	-0.02 ± 0.07	0.2 ± 0.03
NANOG mutant analysis, mutants (GATA6 cell, GATA6 neighbours)	-0.003 ± 0.08	-0.03 ± 0.09	-0.1 ± 0.04

Table 5: Correlation values for Random model 3 for treated embryos (mean ± standard deviation)

Experimental condition	Correlation (no staging)
Treatment analysis, control (NANOG cell, NANOG neighbours)	-0.003 ± 0.03
Treatment analysis, control (GATA6 cell, GATA6 neighbours)	-0.001 ± 0.04
Treatment analysis, PD03 (NANOG cell, NANOG neighbours)	-0.003 ± 0.04
Treatment analysis, PD03 (GATA6 cell, GATA6 neighbours)	-0.005 ± 0.04

4. Rule-based simulations of population composition in ICM of early blastocysts

To generate the simulations of the four populations, we used the 64 early embryo data sets from Saiz et al. For each ICM cell, we determined the simulated cell population type based on two rules and kept the cell centroid and neighbours as obtained from the experimental data. We included the TE cells that are neighbouring at least one ICM cell with their features obtained from the experimental data. To obtain the population type for an ICM cell, we assigned it N+ or N- and G6+ or G6- expression according to these two rules:

1) G6+ cells are clustered; the clustering is achieved by randomly setting the percentage of being G6+ to 85 % and the rest to G6- ($p_{GATA6} = 0.85$);

2) cells with nine or close to nine neighbours are N+ up to 82 % ($p_{NANOG} = 0.82$), the rest N-.

The values for p_{GATA6} and p_{NANOG} are obtained from the experimental data and are the proportion of ICM cells positive for GATA6 or NANOG expression, respectively. Hence, p_{GATA6} is the proportion of DP and N-G+ cells and p_{NANOG} is the proportion of DP and N+G- cells. Combining this information for each cell, we determined its simulated population type.

Sensitivity of the four populations model:

The four populations model relies on three parameters:

1. $p_{GATA6,}$ the proportion of GATA6 positive cells, i.e DP and N-G+ cells
2. p_{NANOG}, the proportion of NANOG positive cells, i.e DP and N+G- cells
3. $\mathrm{startNumNeigh}$, the number of neighbours at which we start assigning NANOG positive fate to the cells

We analysed the sensitivity of the model to the values of these three parameters. We varied p_{NANOG} and p_{GATA6} between 0 and 1 in steps of 0.2 and $\mathrm{startNumNeigh}$ between 7 and 14 in steps of 1. For each parameter value combination, we performed 100 simulations. For each embryo, we calculated the mean distribution of populations of the 100 simulations. The mean population distributions are then summed up to obtain the total overall population distribution. This simulated total population distribution is then compared to the total population distribution from the experimental data. To assess the goodness of fit, we employed the mean squared error $MSE = Mean((\mathrm{popDist}_{Sim} - \mathrm{popDist}_{Exp})^2)$, where $\mathrm{popDist}_{Sim}$ is the population distribution of the simulations and $\mathrm{popDist}_{Exp}$ the population distribution obtained from the experiments. For a better visualisation, we rescale the MSE and obtain the simulation match $1 - \frac{MSE - Min(MSE)}{Max(MSE) - Min(MSE)}$. Hence, a simulation match of 0 corresponds to the parameter values with the worst match and a simulation match of 1 to the best match (Fig 7)

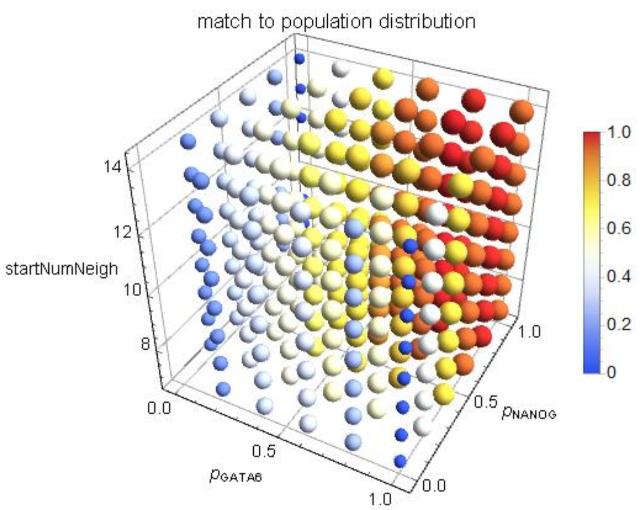

Fig 7: Higher values of p_{NANOG} and p_{GATA6} provide a better match of the simulations to the experimental data. Simulation match to experimental data from early embryos (normalized to [0,1]) for the four populations model, varying p_{NANOG} and p_{GATA6} between 0 and 1 and $\mathrm{startNumNeigh}$ between 7 and 14.

Varying $\mathrm{startNumNeigh}$ independently showed the best match for 14, followed by 9 (Fig 8A). Plotting the population distributions however showed that the differences between the simulations are negligible (Fig 8B).

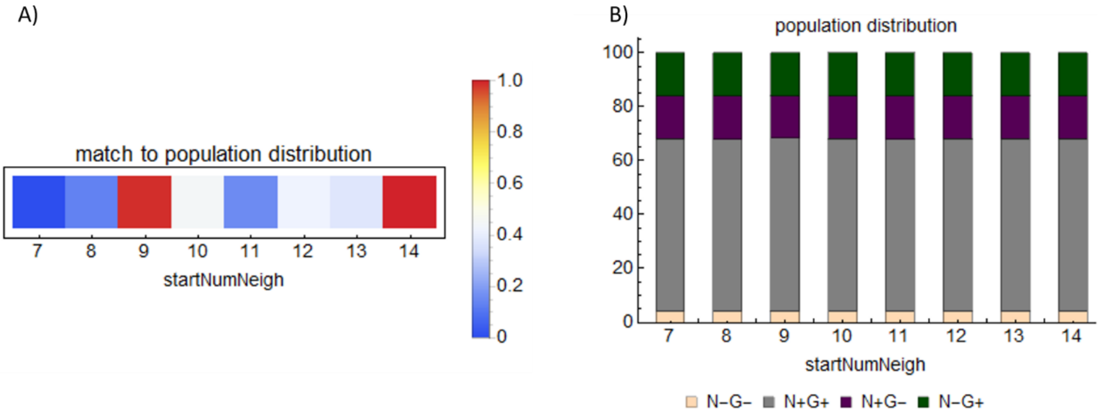

Fig 8: The four populations model is robust with respect to the parameter startNumNeigh. Simulation match to experimental data from early embryos (normalized to [0,1]) (A) and population distributions (B) for the four populations model for $p_{NANOG} = 0.8$ and $p_{GATA6} = 0.8$ and $\mathrm{startNumNeigh}$ varying between 7 and 14.

Varying p_{NANOG} and p_{GATA6} shows that for values between 0.6 and 1 for both parameters, we obtain a reasonable fit, with the best fit for $p_{NANOG} = 0.8$ and $p_{GATA6} = 0.8$ (Fig 9A). Plotting the population distribution for the simulations of these nine parameter combinations, shows that increasing the values for p_{NANOG} or p_{GATA6} changes the composition of the populations in the simulated ICMs (Fig 9B). If p_{NANOG} or p_{GATA6} is one, only up to two populations arise. For values below one for p_{NANOG} and p_{GATA6}, the four populations arise and become more evenly distributed the smaller the values are.

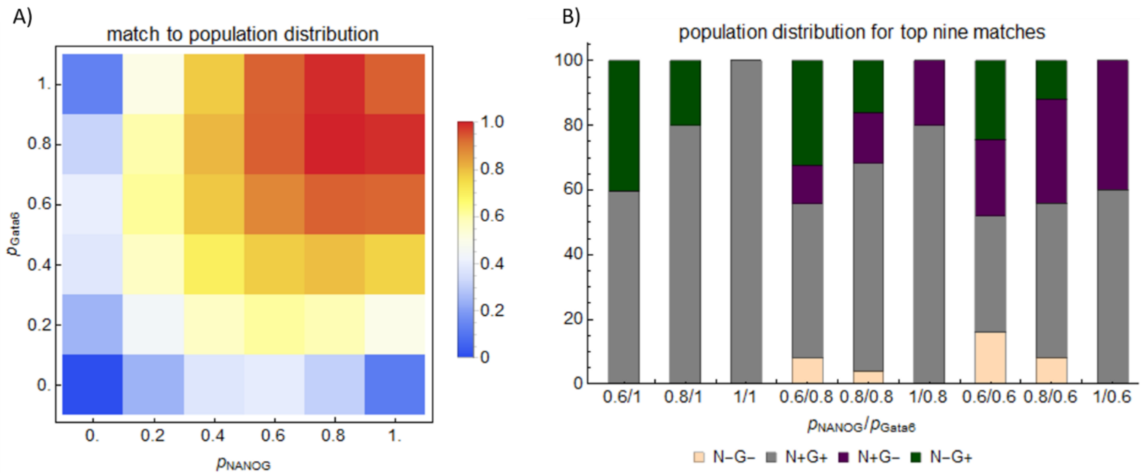

Fig 9: The values of p_{NANOG} and p_{GATA6} determine the population distributions in the simulated ICMs. Simulation match to experimental data from early embryos (normalized to [0,1]) (A) and population distributions for the top nine matches (B) for the four populations model for $\mathrm{startNumNeigh} = 9$ and p_{NANOG} and p_{GATA6} varying between 0 and 1.

References:

1. Saiz N, Williams KM, Seshan VE, Hadjantonakis A-K. Asynchronous fate decisions by single cells collectively ensure consistent lineage composition in the mouse blastocyst. Nat Commun. 2016;7: 13463. doi:10.1038/ncomms13463

2. Schmitz A, Fischer SC, Mattheyer C, Pampaloni F, Stelzer EHK. Multiscale image analysis reveals structural heterogeneity of the cell microenvironment in homotypic spheroids. Sci Rep. 2017;7: 43693. doi:10.1038/srep43693

3. Kaliman S, Jayachandran C, Rehfeldt F, Smith A-S. Limits of Applicability of the Voronoi Tessellation Determined by Centers of Cell Nuclei to Epithelium Morphology. Front Physiol. 2016;7: 551. doi:10.3389/fphys.2016.00551
4. Schaller G, Meyer-Hermann M. Multicellular tumor spheroid in an off-lattice Voronoi-Delaunay cell model. Phys Rev E. 2005;71: 051910. doi:10.1103/PhysRevE.71.051910
5. Lou X, Kang M, Xenopoulos P, Muñoz-Descalzo S, Hadjantonakis AK. A rapid and efficient 2D/3D nuclear segmentation method for analysis of early mouse embryo and stem cell image data. Stem Cell Reports. 2014;2: 382–397. doi:10.1016/j.stemcr.2014.01.010
6. Tosenberger A, Gonze D, Bessonnard S, Cohen-Tannoudji M, Chazaud C, Dupont G. A multiscale model of early cell lineage specification including cell division. npj Syst Biol Appl. 2017;3: 16. doi:10.1038/s41540-017-0017-0
7. Quinn GP, Keough MJ. Experimental Design and Data Analysis for Biologists. 2002 [cited 13 Feb 2018]. Available: http://www.cambridge.org
8. Goodwin LD, Leech NL. Understanding Correlation: Factors That Affect the Size of r. J Exp Educ. 2006;74: 249–266. doi:10.3200/JEXE.74.3.249-266
