# Supplementary material for: The transition from local to global patterns governs the differentiation of mouse blastocysts
Source: PLoS One. 2020 May 15;15(5):e0233030. doi: 10.1371/journal.pone.0233030 (PMC7228118; doi:10.1371/journal.pone.0233030)
Supplement: S1 Fig — Step 1: The confocal images of the fixed embryos are segmented with MINS to obtain the centroid, the cell type (ICM or TE) and the mean NANOG and GATA6 expression levels of a nucleus. Subsequently, the cell fate assignment to TE or ICM is manually checked. Step 2: Data I, provided in four different independently imaged batches, are aligned according to their thresholds for high NANOG and high GATA6 expression levels. Top: Scatter plots showing the raw values for NANOG (horizontal axis) and GATA6 (vertical axis) levels in ICM cells in early, mid and late blastocysts (left, centre and right, respectively) in arbitrary units (a.u.). Each dot represents the levels in a single cell from 26 early, 4 mid and 15 late blastocysts. Further details on the number of embryos and cells analysed are in S1 and S2 Tables. Bottom: Scatter plots showing NANOG (horizontal axis) and GATA6 (vertical axis) levels in ICM cells in early, mid and late blastocysts (left, centre and right, respectively) after aligning the data sets. Dashed lines represent the threshold levels for NANOG and GATA6. Step 3: (i) Illustration of the cell position rescaling for one embryo to account for slight squeezing along the z-axis due to the mounting. (ii) Illustration of the Delaunay Cell Graph (DCG) for this embryo. Lines represent neighbourhood relationship between cells. Step 4: Selecting the cells that are relevant for the analyses. We analyse the features of the ICM cells and as neighbours we include the ICM cells and the TE cells that are neighbouring at least one ICM cell. Illustration of the selected cells and the DCG (left), and of the table containing the relevant data (right). See S1 Sup. Info. text for further details. (PDF) [file pone.0233030.s002.pdf]

Fig. S1

Step 1: Imaging, MINS segmentation, assigning ICM and TE, single cell identification and measurements (NANOG and GATA6 expression levels and cell centroid)

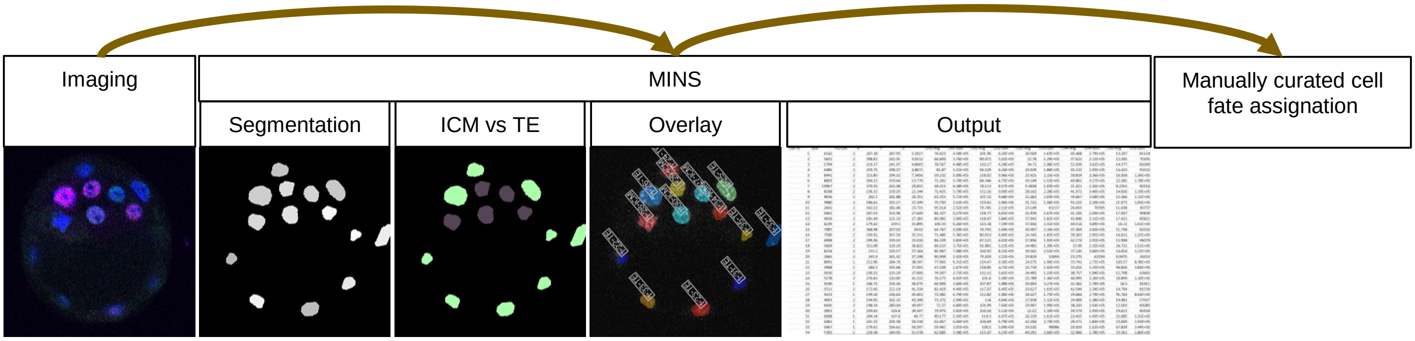

Step 2: Data alignment and assigning cell population type to ICM cells

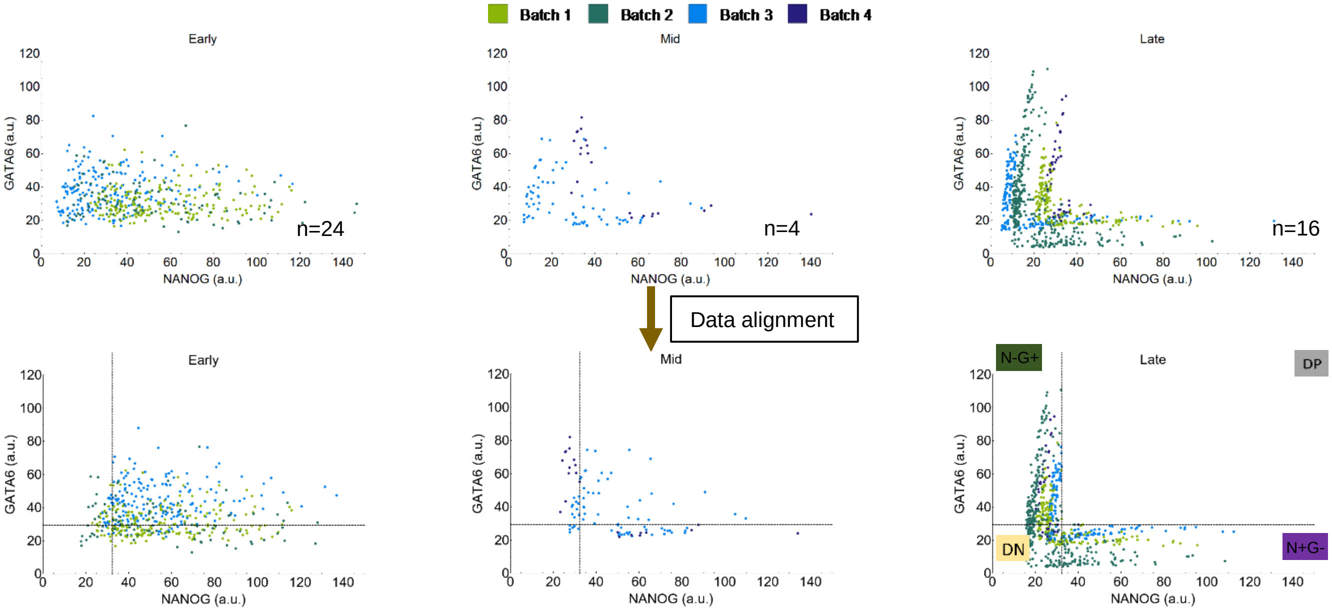

Step 3: Rescaling cell positions to account for mounting (i) and approximation of nearest neighbours by Delaunay triangulation (ii)

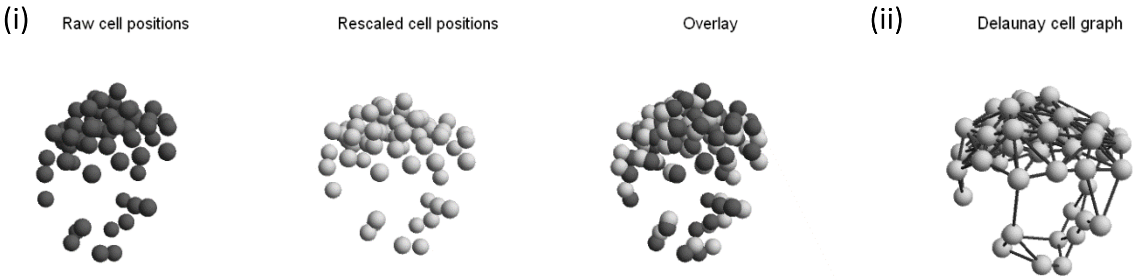

Step 4: Selecting subgraph containing ICM cells and the TE cells that are neighbours to at least one ICM cell

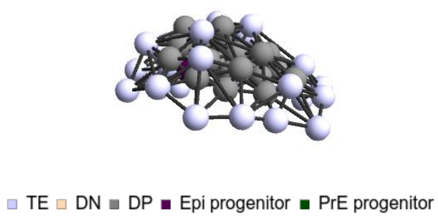

Resulting data for the cells

ID	Centroid	NANOG level	GATA6 level	Population	Neighbour IDs	…
1	{169,214,235}	20	80	N-G+	4, 7, 12, 21	…
2	{173,174,227}	20	30	TE	-	…
3	{179,178,210}	100	20	N+G-	2, 5, 7, 23, 14, 11, 8, 16	…
…	…	…	…	…	…	…
