# Supplementary figures and images for: The transition from local to global patterns governs the differentiation of mouse blastocysts
Source: PLoS One. 2020 May 15;15(5):e0233030. doi: 10.1371/journal.pone.0233030 (PMC7228118; doi:10.1371/journal.pone.0233030)

Fig. S2

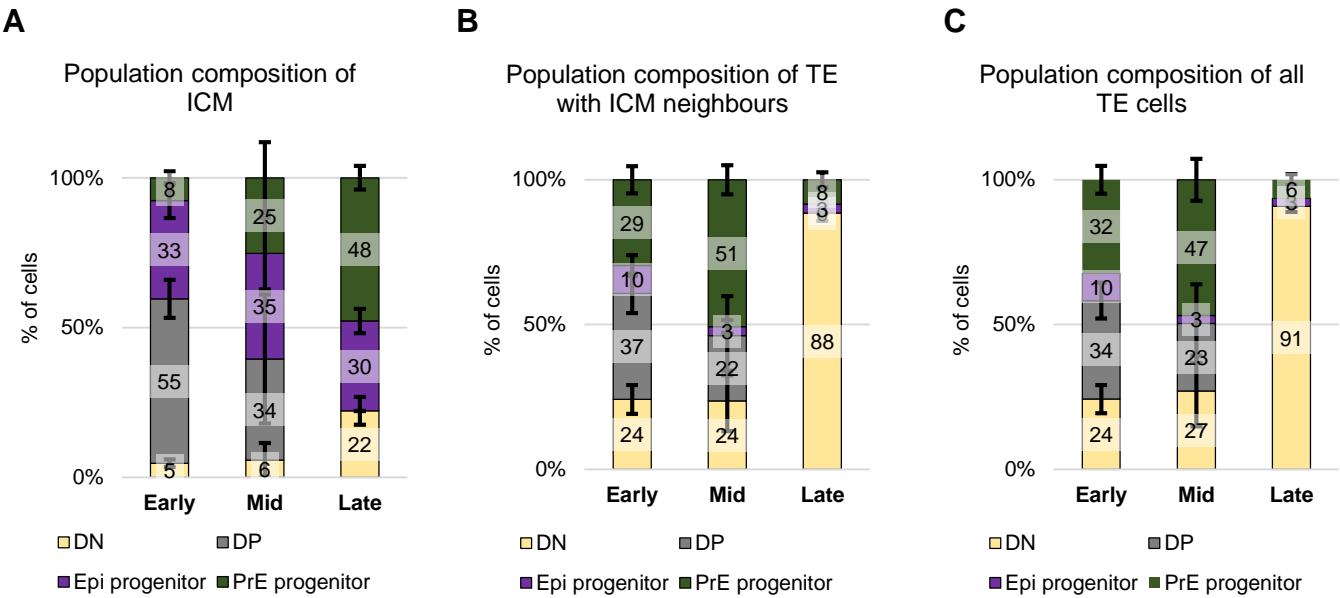

Supplement: S2 Fig — (A-C) Population analysis of individual embryos staged by total cell number (early: 32–64 cells, mid: 65–90 cells, late >90) of all ICM cells (A), TE cells with ICM neighbouring cells (included in subsequent analyses, B) and all TE cells (C). Error bars indicate the standard errors of the means. Details on the number of embryos and cells analysed are in S1 and S2 Tables. (PDF) [file pone.0233030.s003.pdf]

Fig S3:

A

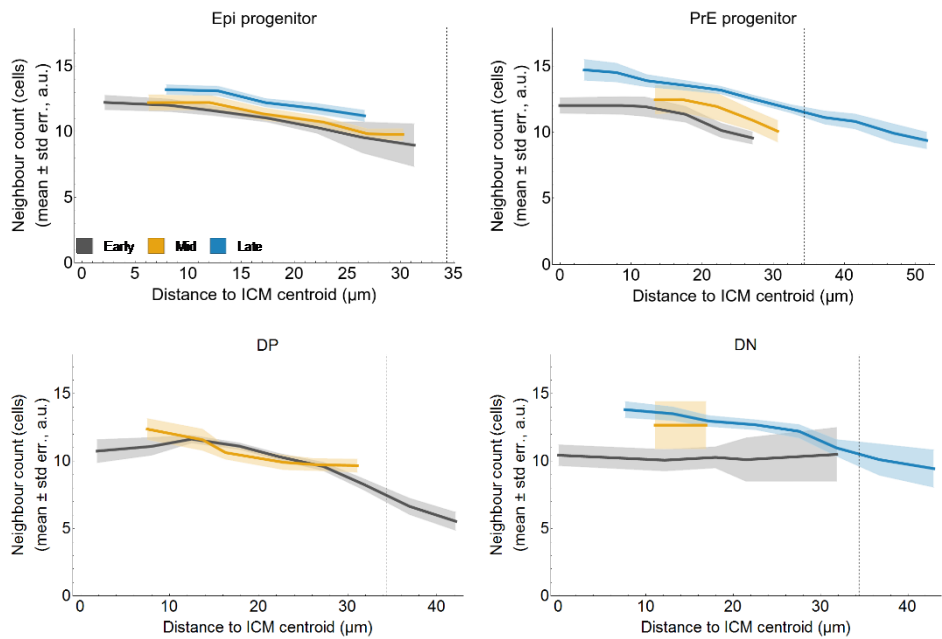

B

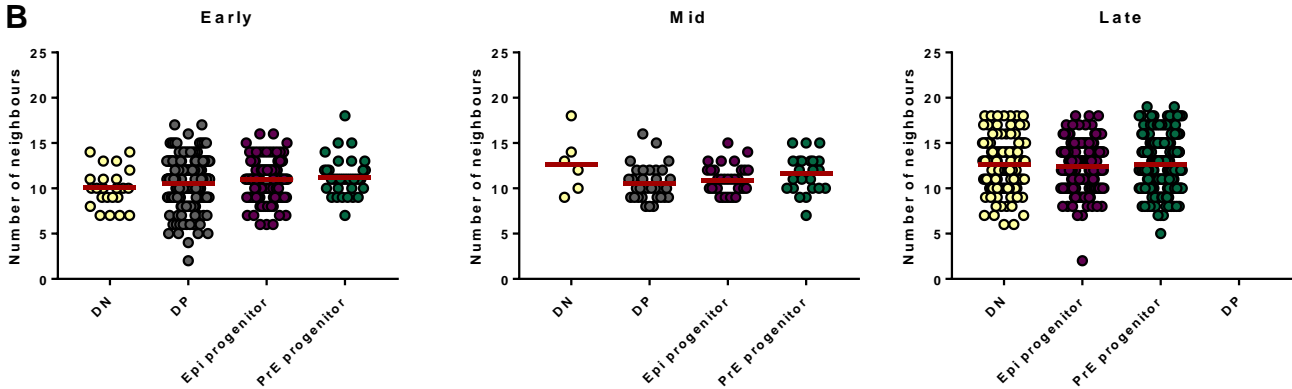

Supplement: S3 Fig — (A) Mean number of neighbouring cells (vertical axis) versus the distance to the ICM centroid (horizontal axis) of the indicated cell populations in ICMs of early (grey), mid (yellow) and late (blue) blastocysts. Shaded regions indicate the standard errors of the means. (B) Scatter dot plot showing the total number of neighbouring cells of DN, DP, Epi progenitor and PrE progenitor cells in ICMs of early (left panel), mid (centre) and late (right) embryos. Mann-Whitney test with Bonferroni correction gives no statistically significant results in all the comparisons (p<0.05). The red horizontal line indicates the mean values. Details on the number of embryos and cells analysed are in S1 and S2 Tables. (PDF) [file pone.0233030.s004.pdf]

**Fig. S5**

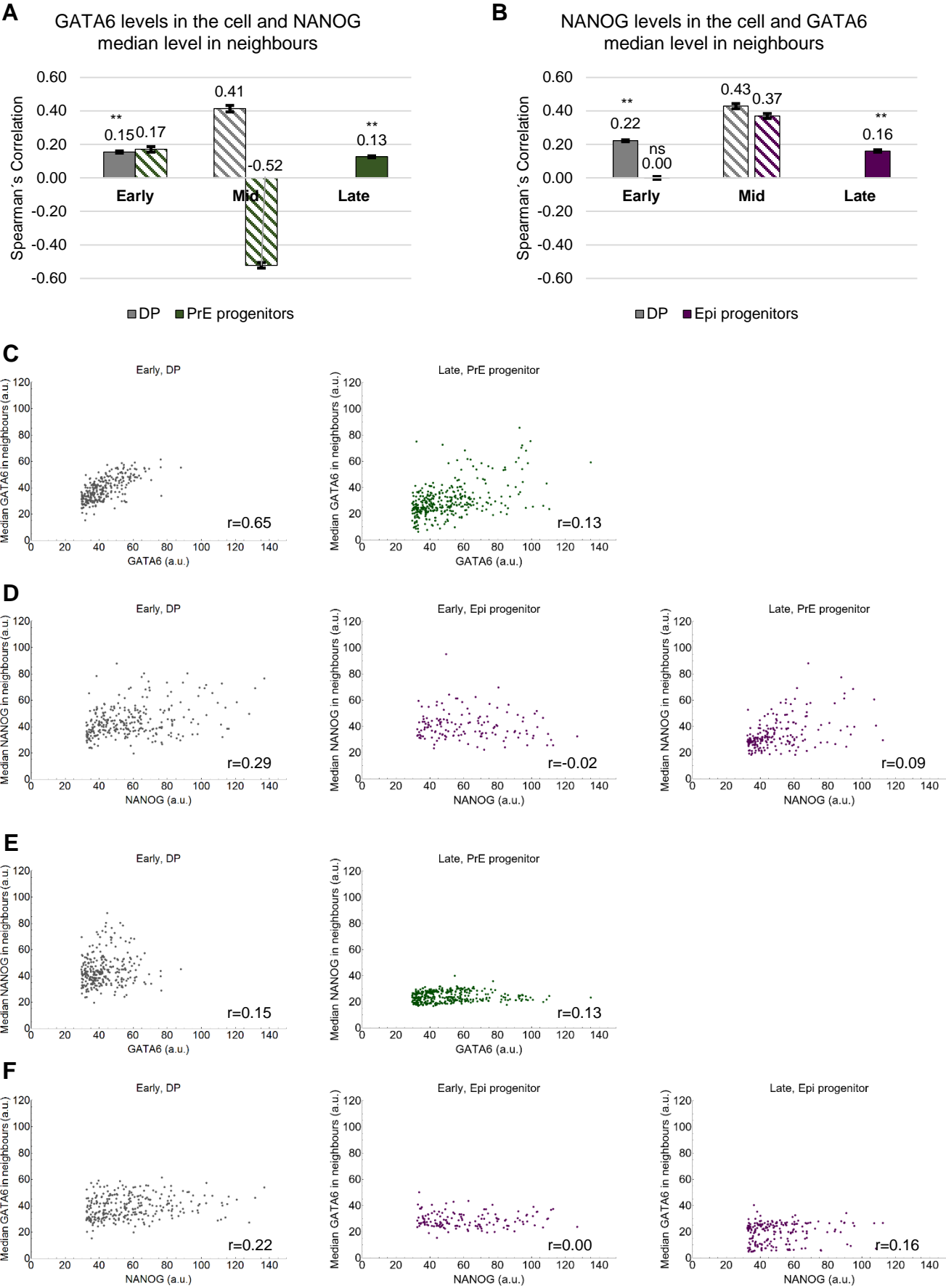

Supplement: S5 Fig — (A-B) Spearman’s correlation coefficients for GATA6 levels of a cell and the median NANOG levels of its neighbours (A) and NANOG levels of a cell and the median GATA6 levels of its neighbours (B) at different embryonic developmental stages. **: p<0.01 Mann-Whitney test with Bonferroni correction for comparison with the null model (see S1 Text for further details). The error bars represent the standard errors calculated by bootstrap sampling the experimental data 100 times. Striped boxes indicate populations composed by less than 108 cells. In those cases, no statistical analysis was performed. (C-F) Scatter dot plots of the expression levels of the indicated fate markers in individual cells (horizontal axis) and the indicated median fate marker levels of their neighbours (vertical axis) in the specified cell population types and developmental stages in arbitrary units (a.u.). Each dot represents a cell. Only those populations composed of more than 108 cells are shown. The Spearman’s correlation coefficients are shown (r). Details on the number of embryos and cells analysed are in S1 and S2 Tables. (PDF) [file pone.0233030.s006.pdf]

Fig. S6

Part 1

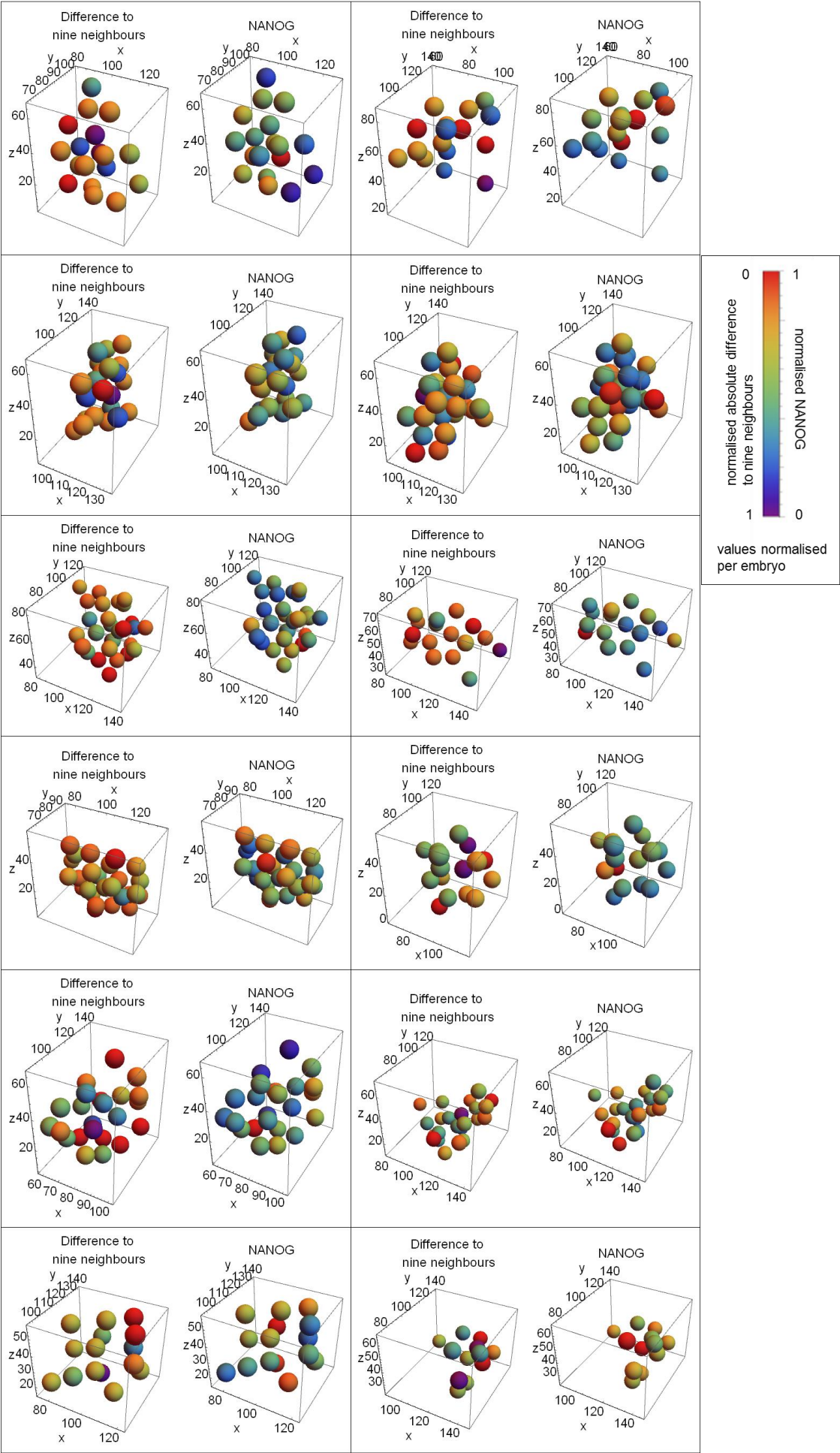

Fig. S6

Part 2

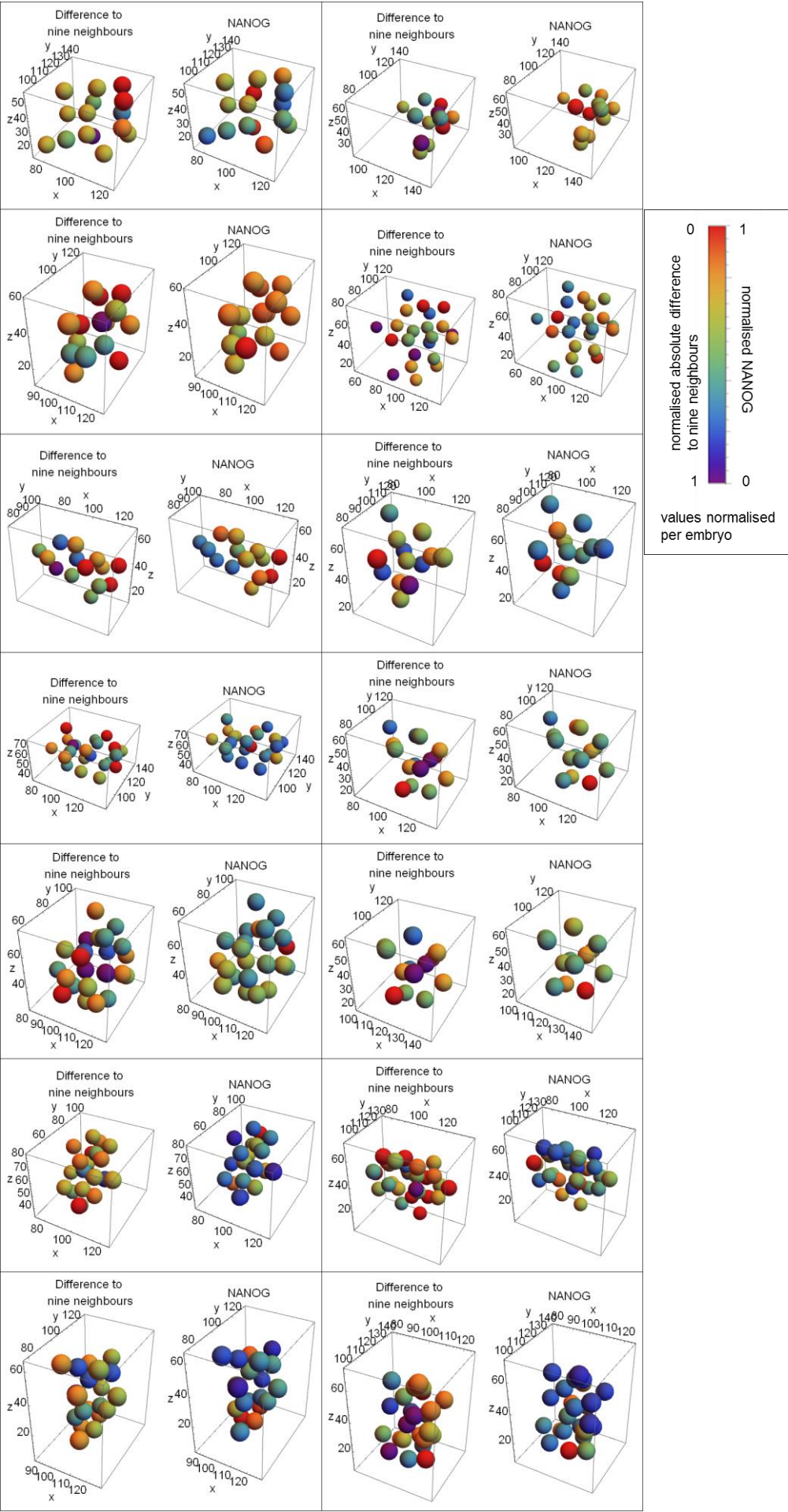

Supplement: S6 Fig — Three-dimensional Illustrations for number of neighbours and NANOG level for all ICM cells in all early blastocysts of data I. For each embryo two illustrations are shown: the normalised absolute difference of the number of neighbours of a cell to nine (left) and the normalised expression level of NANOG (right). Both values are normalised to the maximum in each embryo. I.e. Cells with nine neighbours and maximum NANOG level are shown in red in both images. (PDF) [file pone.0233030.s007.pdf]

**Fig. S8**

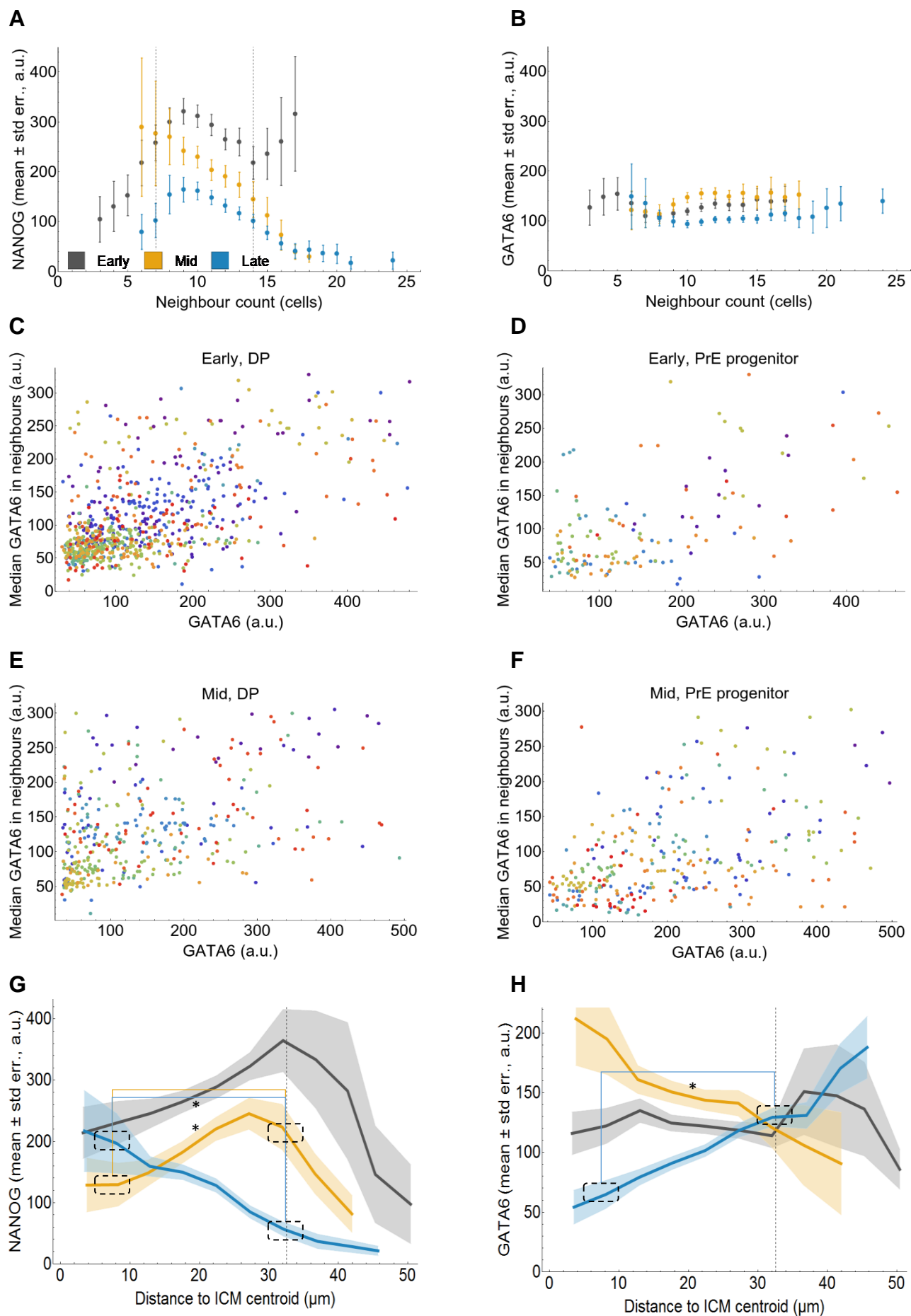

Supplement: S8 Fig — (A, B) Mean level of NANOG (A) or GATA6 (B) (vertical axis) versus the number of neighbours (horizontal axis) for ICM cells in early (grey), mid (yellow) and late (blue) blastocysts. The tail of the graph for early embryos in (A) is due to DP cells with high NANGOG levels and a large number of neighbours. The error bars indicate the standard errors of the means. (C-F) Scatter dot plots of the expression levels of the indicated fate markers in individual cells (horizontal axis) and the indicated median fate marker levels of their neighbours (vertical axis) in the specified cell population types and developmental stages in arbitrary units (a.u.). Each dot represents a cell. The colours represent different embryos. Only those populations with at least a moderate correlation strength, i.e. correlation coefficient greater than 0.4, are shown. (G, H) Mean level of NANOG (G) or GATA6 (H) (vertical axis) versus the distance to the ICM centroid (horizontal axis) for ICM cells in early, mid and late blastocysts. Mann-Whitney test between the indicated levels; **: p<0.05. For simplicity, only selected significant results are indicated for NANOG levels in mid and late embryos, GATA6 levels in late embryos, full statistical results are shown in S9 Fig. The shaded regions indicate the standard errors of the means. Details on the number of embryos and cells analysed are in S1 and S2 Tables. (PDF) [file pone.0233030.s009.pdf]

**Fig. S11**

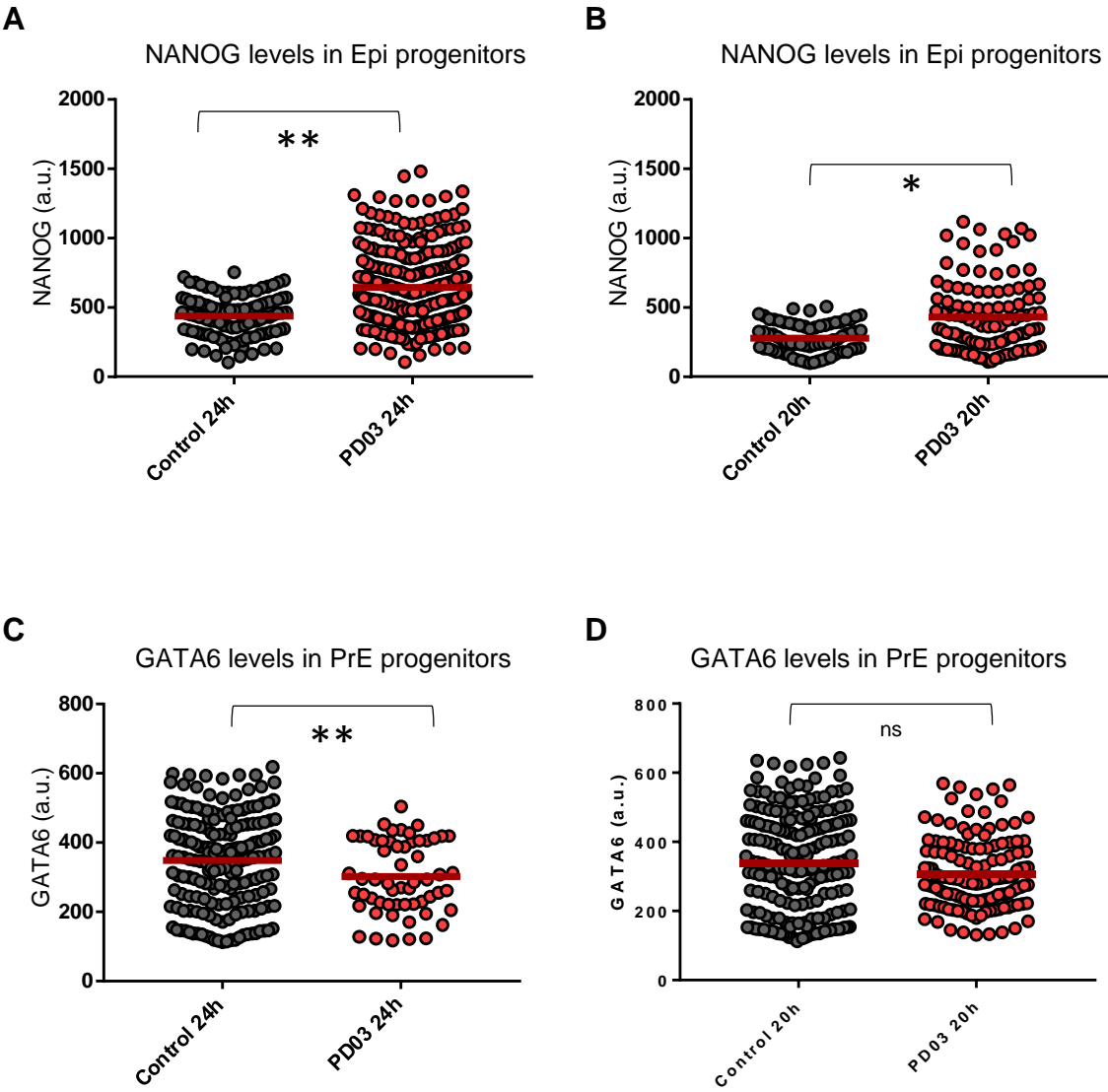

Supplement: S11 Fig — (A-B) Scatter dot plots showing the expression levels of NANOG in Epi progenitor cells of embryos cultured for 24 h (A) or 20 h (B) with control (grey) or PD03-containing (red) media; **: p<0.01 Mann-Whitney test with Bonferroni correction. (C-D) Scatter dot plots showing the expression levels of GATA6 in PrE progenitor cells of embryos treated for 24 h (C) or 20 h (D) with PD03; **: p<0.01 Mann-Whitney test with Bonferroni correction; ns: not significant. In all plots, the red horizontal line indicates the mean values. Details on the number of embryos and cells analysed are in S1 and S2 Tables. (PDF) [file pone.0233030.s012.pdf]

**Fig. S12**

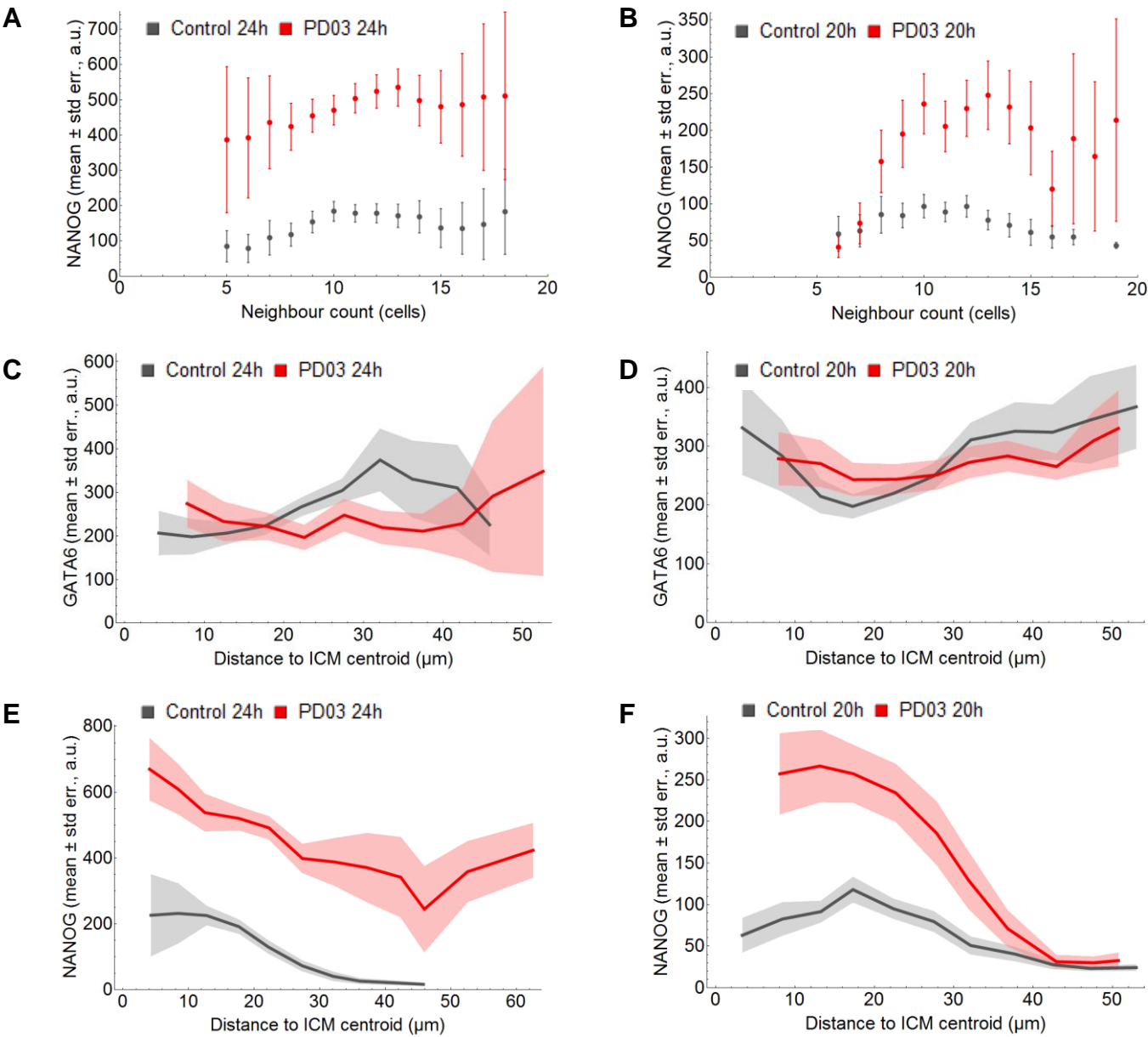

Supplement: S12 Fig — (A-B) Mean level of NANOG (vertical axis) versus the number of neighbours (horizontal axis) for ICM cells in embryos cultured for 24 h (A) or 20 h (B) with control (grey) or PD03-containing (red) media. (C-D) Mean level of GATA6 (vertical axis) versus the distance to the centre of the ICM (horizontal axis) for ICM cells in embryos treated for 24 h (C) or 20 h (D) with PD03. (E-F) Mean level of NANOG (vertical axis) versus the distance to the centre of the ICM (horizontal axis) for ICM cells in embryos treated for 24 h (E) or 20 h (F) with PD03. In all plots, error bars or shaded regions indicate the standard errors of the means, respectively. Details on the number of embryos and cells analysed are in S1 and S2 Tables. (PDF) [file pone.0233030.s013.pdf]
