# Supplementary material for: The transition from local to global patterns governs the differentiation of mouse blastocysts
Source: PLoS One. 2020 May 15;15(5):e0233030. doi: 10.1371/journal.pone.0233030 (PMC7228118; doi:10.1371/journal.pone.0233030)
Supplement: S4 Fig — Tables showing the statistical test results (z-test) for a pairwise comparison of cell neighbour type for each cell population type in the different developmental stages for data set I. *: p<0.05 (with Bonferroni correction); ns: not significant. E.g. a DN cell has significantly more TE neighbours than DN neighbours. Details on the number of embryos and cells analysed are in S1 and S2 Tables. (PDF) [file pone.0233030.s005.pdf]

Fig S4:

Early Blastocyst		Neighbour population type												
		TE vs DN	TE vs DP	TE vs Epi prog.	TE vs PrE prog.		DN vs DP	DN vs Epi prog.	DN vs PrE prog.		DP vs Epi prog.	DP vs PrE prog.		Epi prog. vs PrE prog.
Cell type	DN cell	*	*	*	*		*	*	ns		ns	*		*
	DP cell	*	*	*	*		*	*	*		*	*		*
	Epi prog. cell	*	*	ns	*		*	*	*		*	*		*
	PrE prog. cell	*	*	*	*		*	*	*		ns	*		*

Mid Blastocyst		Neighbour population type												
		TE vs DN	TE vs DP	TE vs Epi prog.	TE vs PrE prog.		DN vs DP	DN vs Epi prog.	DN vs PrE prog.		DP vs Epi prog.	DP vs PrE prog.		Epi prog. Vs PrE prog.
Cell type	DN cell	ns	*	ns	*		*	ns	ns		*	ns		ns
	DP cell	*	*	*	*		*	*	*		*	*		ns
	Epi prog. cell	*	*	ns	*		ns	*	*		*	*		*
	PrE prog. cell	*	*	*	*		ns	*	*		*	*		ns

Late Blastocyst		Neighbour population type							
		TE vs DN	TE vs Epi prog.	TE vs PrE prog.		DN vs Epi prog.	DN vs PrE prog.		Epi prog. Vs PrE prog.
Cell type	DN cell	ns	*	*		*	*		ns
	Epi prog. cell	*	*	*		*	ns		*
	PrE prog. cell	*	*	ns		ns	*		*
