# Supplementary material for: The transition from local to global patterns governs the differentiation of mouse blastocysts
Source: PLoS One. 2020 May 15;15(5):e0233030. doi: 10.1371/journal.pone.0233030 (PMC7228118; doi:10.1371/journal.pone.0233030)
Supplement: S7 Fig — (A) Mean level of NANOG (left) or GATA6 (right) (vertical axis) versus the number of neighbours (horizontal axis) for the null model simulation of ICM cells of data I in early (grey), mid (yellow) and late (blue) blastocysts. Error bars indicate the standard errors of the means. (B) Mean level of NANOG (left) or GATA6 (right) (vertical axis) versus the distance to the ICM centroid (horizontal axis, binned in 5 μm groups) for the null model simulation of ICM cells of data I in early (grey), mid (yellow) and late (blue) blastocysts. Shaded regions indicate the standard errors of the means. (C) Tables summarizing the statistically significant results of the Mann-Whitney statistical tests with Bonferroni correction comparing NANOG or GATA6 levels at the indicated positions relative to the ICM centroid; *: p<0.05. Related to Fig 4C and 4D. (PDF) [file pone.0233030.s008.pdf]

Fig. S7

A

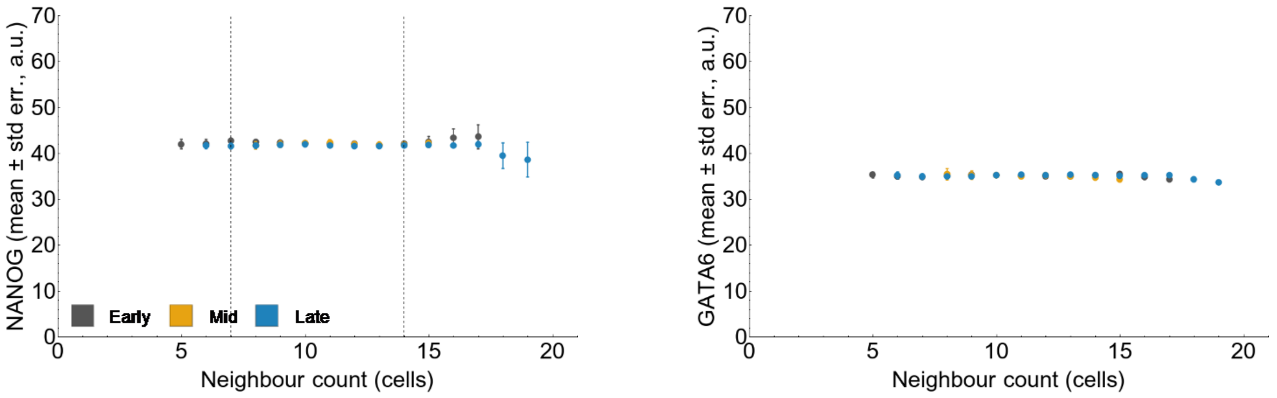

B

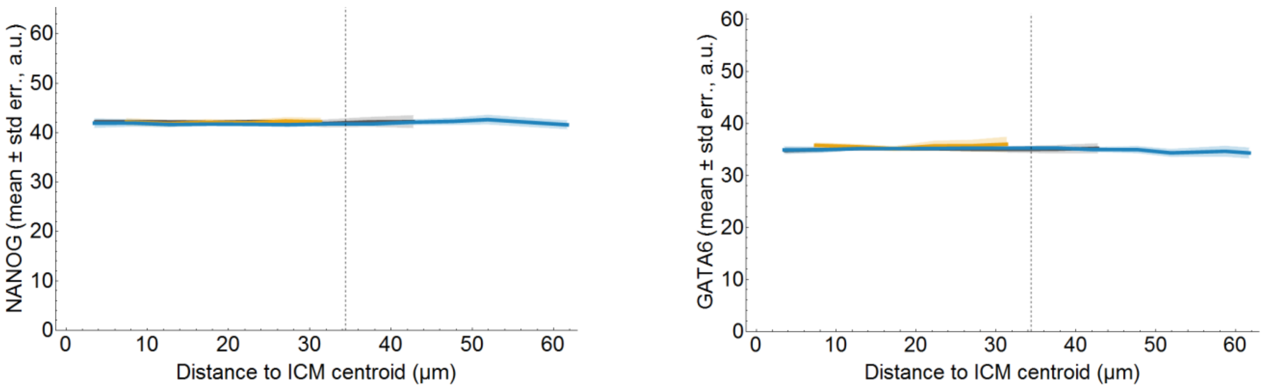

C

NANOG levels at position (late blastocysts)	25 µm-29.9 µm	30 µm-34.9 µm	35 µm-39.9 µm	40 µm-44.9 µm
10 µm-14.9 µm	*	*	*	*
15 µm-19.9 µm	ns	*	*	*
20 µm-24.9 µm	ns	*	*	*
2 5µm-29.9 µm		ns	*	*

GATA6 levels at position (late blastocysts)	25 µm-29.9 µm	30 µm-34.9 µm	35 µm-39.9 µm	40 µm-44.9 µm	45 µm-49.9 µm
5 µm-9.9 µm	ns	ns	*	ns	ns
10 µm-14.9 µm	*	*	*	*	*
15 µm-19.9 µm	ns	*	*	ns	ns
20 µm-24.9 µm	ns	ns	*	ns	ns
25 µm-29.9 µm		ns	*	ns	ns
