# Supplementary material for: The transition from local to global patterns governs the differentiation of mouse blastocysts
Source: PLoS One. 2020 May 15;15(5):e0233030. doi: 10.1371/journal.pone.0233030 (PMC7228118; doi:10.1371/journal.pone.0233030)
Supplement: S9 Fig — Tables summarizing the results of the Mann-Whitney statistical tests with Bonferroni correction comparing NANOG (A-B) and GATA6 (C) levels at the indicated positions relative to the ICM centroid in mid (A) and/or late blastocysts (B-C); *: p<0.05, ns: not significant. Related to S8G and S8H Fig. Details on the number of embryos and cells analysed are in S1 and S2 Tables. (PDF) [file pone.0233030.s010.pdf]

Fig. S9

A

NANOG levels at position (mid blastocysts)	25 µm-29.9 µm	30 µm-34.9 µm
5 µm-9.9 µm	*	*
10 µm-14.9 µm	*	*

B

NANOG levels at position (late blastocysts)	25 µm-29.9 µm	30 µm-34.9 µm	35 µm-39.9 µm
0 µm-4.9 µm	ns	*	*
5 µm-9.9 µm	*	*	*
10 µm-14.9 µm	*	*	*
15 µm-19.9 µm	*	*	*
20 µm-24.9 µm	*	*	*
25 µm-29.5 µm		*	ns

C

GATA6 levels at position (late blastocysts)	25 µm-29.9 µm	30 µm-34.9 µm	35 µm-39.9 µm
10 µm-14.9 µm	ns	*	*
15 µm-19.9 µm	*	*	*
20 µm-24.9 µm	ns	*	*
