# Supplementary material for: The transition from local to global patterns governs the differentiation of mouse blastocysts
Source: PLoS One. 2020 May 15;15(5):e0233030. doi: 10.1371/journal.pone.0233030 (PMC7228118; doi:10.1371/journal.pone.0233030)
Supplement: S10 Fig — (A) Scatter dot plot showing GATA6 expression levels in the indicated cell populations and developmental stages in Nanog+/+ or Nanog+/- and Nanog-/-; **: p<0.01 Mann-Whitney test with Bonferroni correction. The red horizontal line indicates the mean values. (B) Table summarizing the results of the Mann-Whitney statistical tests with Bonferroni correction comparing GATA6 levels at the indicated positions relative to the ICM centroid in Nanog+/+ or Nanog+/- embryos at the indicated positions; *: p<0.05, ns: not significant. Details on the number of embryos and cells analysed are in S1 and S2 Tables. (PDF) [file pone.0233030.s011.pdf]

Fig. S10

A

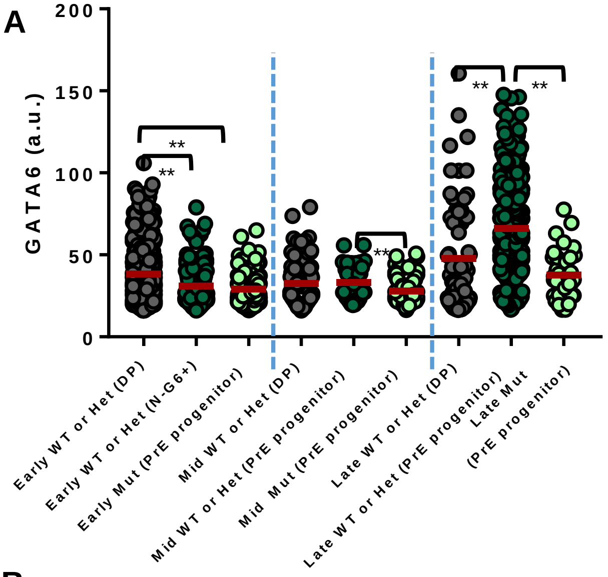

B

GATA6 levels at position (late blastocysts) *Nanog*$^{+/+}$	25 µm-29.9 µm	30 µm-34.9 µm	45 µm-49.9 µm
0 µm-4.9 µm	ns	*	ns
5 µm-9.9 µm	*	ns	ns
10 µm-14.9 µm	*	*	ns
15 µm-19.9 µm	*	ns	ns
25 µm-29.5 µm		ns	*
30 µm-34.9 µm	ns	ns	*
