# Supplementary material for: The transition from local to global patterns governs the differentiation of mouse blastocysts
Source: PLoS One. 2020 May 15;15(5):e0233030. doi: 10.1371/journal.pone.0233030 (PMC7228118; doi:10.1371/journal.pone.0233030)
Supplement: S1 Table — (PDF) [file pone.0233030.s016.pdf]

Sup. Table 1: Number of analysed embryos

Data		Number of embryos			Number of cells in embryos			Number of cells in ICM			Number of cells in analysis (ICM+TE neighbours)			Mean number per DCG		
		early	mid	late	early	mid	late	early	mid	late	early	mid	late	early	mid	late
I	Our WT	24	4	16	1124	302	2329	485	96	712	994	203	1638	41.42	50.75	102.38
II	Saiz *et al* WT	64	34	49	3033	2596	5903	1157	918	1994	2487	1824	4089	38.86	53.65	83.45
III	*Nanog*$^{+/+}$ or *Nanog*$^{+/-}$	44	7	22	2159	493	2779	830	167	860	1901	377	2061	43.20	53.86	93.68
IV	*Nanog*$^{-/-}$	12	5	2	657	348	207	251	132	46	582	280	125	48.50	56.00	62.50
V	treatment control (24 h)	0	1	15	0	81	1735	0	25	449	0	32	852	0	32.00	56.80
VI	treatment control (20 h)	0	1	12	0	74	1329	0	20	435	0	36	946	0	36.00	78.83
VII	treatment PD03 (24 h)	0	0	11	0	0	1194	0	0	447	0	0	657	0	0.00	59.73
VIII	treatment PD03 (20 h)	0	1	7	0	74	740	0	46	292	0	74	497	0	74.00	71.00
