# Supplementary material for: The transition from local to global patterns governs the differentiation of mouse blastocysts
Source: PLoS One. 2020 May 15;15(5):e0233030. doi: 10.1371/journal.pone.0233030 (PMC7228118; doi:10.1371/journal.pone.0233030)
Supplement: S2 Table — (PDF) [file pone.0233030.s017.pdf]

Sup. Table 2: Number of analysed cells

Data		early				mid				late			
		DN	DP	Epi prog.	PrE prog.	DN	DP	Epi prog.	PrE prog.	DN	DP	Epi prog.	PrE prog.
I	Our WT	23	280	145	37	6	34	33	23	170	0	201	341
II	Saiz *et al* WT	37	841	133	146	20	429	138	331	252	200	524	1018
III	*Nanog*$^{+/+}$ or *Nanog*$^{+/-}$	26	627	27	150	6	90	32	39	153	66	198	443
IV	*Nanog*$^{-/-}$	12	5	0	234	2	0	0	130	3	5	0	38
V	treatment control (24 h)	0	0	0	0	1	2	9	13	82	8	151	208
VI	treatment control (20 h)	0	0	0	0	0	0	8	12	84	6	108	237
VII	treatment PD03 (24 h)	0	0	0	0	0	0	0	0	18	25	323	81
VIII	treatment PD03 (20 h)	0	0	0	0	0	0	12	34	9	26	128	129
